# Hydroxymethanesulfonate and Sulfur(IV) in Fairbanks Winter During the ALPACA Study

**DOI:** 10.1021/acsestair.4c00012

**Published:** 2024-05-15

**Authors:** Kayane Dingilian, Elliana Hebert, Michael Battaglia, James R. Campbell, Meeta Cesler-Maloney, William Simpson, Jason M. St. Clair, Jack Dibb, Brice Temime-Roussel, Barbara D’Anna, Allison Moon, Becky Alexander, Yuhan Yang, Athanasios Nenes, Jingqiu Mao, Rodney J. Weber

**Affiliations:** † School of Earth and Atmospheric Sciences, Georgia Institute of Technology, Atlanta, Georgia 30332, United States; ‡ Geophysical Institute and Department of Chemistry & Biochemistry, University of Alaska Fairbanks, Fairbanks, Alaska 99775, United States; § Atmospheric Chemistry and Dynamics Laboratory, NASA Goddard Space Flight Center, Greenbelt, Maryland 20771, United States; ¶ Joint Center for Earth Systems Technology, University of Maryland Baltimore County, Baltimore, Maryland 21228, United States; ∥ Institute for the Study of Earth, Oceans, and Space, University of New Hampshire, Durham, New Hampshire 03824, United States; ⊥ Aix Marseille Univ, CNRS, LCE, Marseille 13003, France; # Department of Atmospheric Sciences, University of Washington, Seattle, Washington 98195, United States; ⊗ Laboratory of Atmospheric Processes and their Impacts, School of Architecture, Civil and Environmental Engineering, École Polytechnique Fédérale de Lausanne, Lausanne 1015, Switzerland; ○ Center for the Study of Air Quality and Climate Change, Institute of Chemical Engineering Sciences, Foundation for Research and Technology Hellas, Patras 26504, Greece

**Keywords:** Arctic pollution, air quality, particulate matter, sulfur, aqueous, supper-cooled drops, hydroxymethanesulfonate

## Abstract

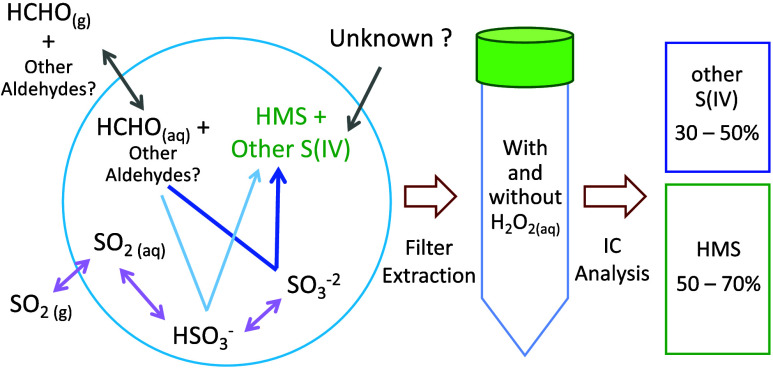

Hydroxymethanesulfonate (HMS) in fine aerosol particles has been reported at significant concentrations along with sulfate under extreme cold conditions (-35 °C) in Fairbanks, Alaska, a high latitude city. HMS, a component of S(IV) and an adduct of formaldehyde and sulfur dioxide, forms in liquid water. Previous studies may have overestimated HMS concentrations by grouping it with other S(IV) species. In this work, we further investigate HMS and the speciation of S(IV) through the Alaskan Layered Pollution and Chemical Analysis (ALPACA) intensive study in Fairbanks. We developed a method utilizing hydrogen peroxide to isolate HMS and found that approximately 50% of S(IV) is HMS for total suspended particulates and 70% for PM_2.5_. The remaining unidentified S(IV) species are closely linked to HMS during cold polluted periods, showing strong increases in concentration relative to sulfate with decreasing temperature, a weak dependence on particle water, and similar particle size distributions, suggesting a common aqueous formation process. A portion of the unidentified S(IV) may originate from additional aldehyde-S(IV) adducts that are unstable in the water-based chemical analysis process, but further chemical characterization is needed. These results show the importance of organic S(IV) species in extreme cold environments that promote unique aqueous chemistry in supercooled liquid particles.

## Abstract 2

There are few studies on fine aerosol particle formation in extreme cold and dark environments. Here we investigate the production of sulfur compounds through reactions with aldehydes in liquid drops under these unique conditions.

## 1 Introduction

Fine particulate matter (i.e., PM_2.5_), a major component of air pollution, is a collection of chemical species linked to adverse effects on human health and the environment.^1,2^ Primary and secondary inorganic and organic sulfur species are a significant portion of PM_2.5_ in many regions.^3^ Aerosol sulfur species affect the hygroscopic properties of particles, influencing aerosol liquid water content (ALWC), aerosol optical properties and cloud condensation nuclei (CCN). These effects may influence climate by directly changing the radiative balance or indirectly by modulating clouds.^4,5^ Sulfur species are also a major contributor to particle acidity, which affects aerosol chemistry and composition,^6^ ecosystem productivity^7^ and human health.^8−10^

Gas phase sulfur dioxide (SO_2_) is a precursor of aerosol particle secondary sulfur. Upon uptake by aqueous drops, it forms inorganic S(IV) species, including dissolved SO_2_ (SO_2_–H_2_O), bisulfite (HSO_3_^–^), and sulfite (SO_3_^2–^) (see reactions R1-R3 below). In fogs, clouds, and aerosol particle water, S(IV) can form other sulfur compounds, such as sulfuric acid (H_2_SO_4_), bisulfate (HSO_4_^–^) and sulfate (SO_4_^2–^).^3^ S(IV) species can also react with aldehydes to produce a range of aldehyde-S(IV) adducts (hydroxyalkylsulfonates, HAS), with hydroxymethanesulfonate (HMS) being the most prominent.^11,12^ HMS is an adduct of aqueous formaldehyde (HCHO) and sulfur dioxide (SO_2_), in the form of sulfite (SO_3_^2–^) or bisulfite (HSO_3_^–^). The basic formation mechanism is
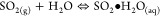
R1
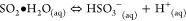
R2
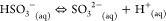
R3
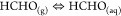
R4
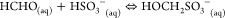
R5
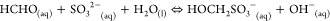
R6

Early studied investigated the HMS formation mechanism through field^13^ and laboratory^14,15^ studies. Boyce and Hoffman^16^ found enhanced reactivity of SO_3_^2–^ with HCHO compared to HSO_3_^–^, making (R6) the dominant HMS formation pathway. Conditions favorable to HMS production and stability were found to be high ambient concentrations of SO_2_ and HCHO,^16^ low temperatures,^15^ a pH of the aqueous environment of approximately 4 to 5, and low concentrations of oxidants that deplete HMS.^3^

Measuring HMS has been challenging due to similarity with sulfate, interference from other S(IV) species during analysis and loss from filter samples over time. Moch et al.^17,18^ noted that these factors may lead to overestimation of sulfate and undermeasurement of HMS when standard measurement protocols are applied to network samples, such as IMPROVE, implying that HMS may be more prevalent than currently believed. Dovrou et al.^19^ used the Dionex AS12A column with pH 3 eluent to separate HMS from SO_4_^2–^ but noted it could not be distinguished from bisulfite. Aerosol Mass Spectrometry (AMS) utilizing thermal desorption also has limitations in identifying HMS in a mixture with other sulfur species (e.g., S(VI)).^19^ Single particle laser ablation Aerosol Time-Of-Flight Mass Spectrometer (ATOFMS) can detect HMS but requires ancillary information to quantify mass concentrations.^20^

Ion chromatographic analysis can be highly quantitative and capable of separating HMS from sulfate, but as noted, co-elution of HMS with other S(IV) species results in overestimation of HMS. Methods to separate HMS from other S(IV) species have not been commonly utilized in recent studies but were in the past. Rao and Collett^21^ quantified the contribution of HMS to S(IV) in cloud and fog water using treatments with hydrogen peroxide to eliminate (through oxidation) inorganic S(IV), assuming no effect on HMS. They found that most S(IV) was present as HMS for large drops (D_p_>35 μm), but to a lesser extent (∼60%) for drops between 3.5 and 35 μm diameter. This method was adapted by Dixon and Aasen^22^ to measure HMS and S(IV) in aerosol samples; they found HMS was only a very small fraction of total aerosol mass.

Recent studies have again investigated HMS. Low fractions of HMS in PM_10_ were observed in Europe,^23^ whereas high concentrations of PM_2.5_ HMS have been measured over the North China Plain (NCP) during cold periods.^24^ HMS concentrations of up to 7.3 μg/m^3^ (avg 2.0 ± 2.1 μg/m^3^) in Beijing, have been associated with fog.^25^ Some rural sites may have higher HMS concentrations than urban areas because of heightened fog and precursor concentrations.^26^ There is also evidence of HMS formation in fine particle liquid water.^24,27^ In these regions, including HMS in model predictions produced better agreement with observations of sulfur species concentrations.^17,28^

We recently reported on another region of high concentrations of HMS (actually S(IV)).^29^ In Fairbanks, Alaska, during extremely cold periods (-35°C), that accompanied sever pollution events. We found S(IV) (which was referred to as HMS) comprised 26-41% of particulate sulfur by mass and 2.8-6.8% of overall PM_2.5_ mass. These conditions are in stark contrast to those under which atmospheric aqueous reactions are typically investigated and a significantly colder environment than the studies of HMS over the NCP. HMS only forms in liquid water, its formation in the extreme cold of Fairbanks may be possible due to freezing point depression effects^30^ from the salts present in the particles.

In this study, we extend the analysis of HMS in Fairbanks winter by developing methods to separate HMS from other S(IV) species to quantify components of S(IV). We identify factors that promote HMS and S(IV) formation and utilize particle size-resolved measurements of HMS and S(IV) to better understand the sources and atmospheric processes affecting PM_2.5_ concentrations during pollution event in wintertime Fairbanks, Alaska. Our findings shed light on unique atmospheric chemistry in extremely cold environments, both in Alaska and similar environments worldwide.

## 2 Materials and Methods

### 2.1 Sampling Location

The Alaskan Layered Pollution and Chemical Analysis (ALPACA) collaborative study was conducted from 17 January to 25 February 2022 with measurement sites at several locations in the city of Fairbanks, Alaska (latitude 64.84°N). Measurements included meteorological parameters,^31^ a suite of gas-phase aldehydes, and online and offline methods to determine particle chemical composition at a site adjacent to the University of Alaska Fairbanks Community and Technical College in downtown Fairbanks (UAF CTC, 64.84°N, 147.73°W, elevation 136 m above sea level). Hourly measurements of PM_2.5_ mass concentration (Met-One BAM 1020X), and sulfur dioxide (SO_2_) (Thermo Scientific 43iQ-TL) concentrations were made by the Alaska Department of Environmental Conservation (ADEC) at the NCore site about 500 m from the CTC site.

Pollution events in Fairbanks are characterized by large increases in PM_2.5_ mass concentration and primary species, such as carbon monoxide (CO), nitrogen oxides (NOx) and SO_2,_ but low concentrations of ozone (O_3_) due to limited sunlight and titration by NOx. These events occur during periods of very low temperatures, stable atmospheric conditions with calm winds and strong shallow temperature inversions that limit dispersion of the urban emissions.^32,33^

### 2.2 Analytical Methods

In all cases, sample inlets were typically 3 to 5 m above ground level. The main instruments and analytical methods used are described below. All particle concentrations reported are at ambient temperature and pressure and all date/times are Alaska Standard Time (AKST or local time in winter), which is Coordinated Universal Time (UTC) minus 9 h.

#### 2.2.1 Online Anions with a PILS-IC

For online measurements of PM_2.5_ inorganic chemical species concentrations, including S(IV), we used the particle-into-liquid sampler–ion chromatography (PILS-IC) system.^29,34^ Ambient air at nominally 16.7 L/min passed through a sharp cut cyclone (URG-2000-30EH, URG Corporation) followed by two denuders - parallel plate activated carbon^35^ and sodium carbonate-coated etched glass honeycomb to remove acidic gases–all at ambient temperature. The sample line entered the heated building (nominally 20 to 25°C) and directly into the PILS through a 5 cm long thermally insulated tube. All sample lines were 1.27 cm outer diameter and 1.08 cm inner diameter stainless steel tubing. Sample water exiting the PILS containing the dissolved PM_2.5_ species was combined with a transport flow spiked with lithium bromide to determine the total sample liquid flow rate. The combined liquid sample was continuously pumped through a 250 μL sample loop and injected every 23 min into the separation column, the time required to achieve chromatographic separation for this setup. The analysis was performed with an anion IC unit for the entire study period. The IC unit, column, eluent and operational parameters for online and offline analyses are summarized below (IC Analytical Methods). The PILS-IC system was capable of separating S(IV) and S(VI) (SO_4_^2–^) but was not configured to isolate HMS, a component of S(IV). The detection limits for S(IV) and sulfate were 0.15 μg/m^3^ and 0.01 μg/m^3^, respectively, with an estimated uncertainty of 30%, mainly due to variable dilution by vapor condensation in the PILS at the extreme low temperatures of Fairbanks.^29^

#### 2.2.2 Filter Collection and Storage

Three filter collection systems located outdoors at ambient conditions were deployed at the CTC site.

##### 2.2.2.1 Georgia Institute of Technology (GT)

A Teflon filter (Whatman, 47 mm diameter, 1 μm pore size) was installed downstream of a 2.5 μm URG sharp-cut cyclone and parallel plate activated carbon denuder.^35^ The sample flow rate was nominally 16.7 L/min ±10%. All sample lines were 1.27 cm diameter stainless steel. Samples were replaced once daily at approximately 10:00 and measured through 9:00 AKST the next day.

##### 2.2.2.2 University of Washington (UW)

A high-volume sampler (1.42 m^3^/min) separated particles into five size ranges: < 0.7 μm, 0.7-1.1 μm, 1.1-2.5 μm, 2.5-5.8 μm, and 5.8-10 μm aerodynamic diameter.^36^ Particles were captured with quartz filters (TE-QMA and TE-230-QZ) that were collected daily at nominally the same schedule as the GT filters, except for twice-daily samples during the cold pollution event of high PM_2.5_ mass concentration (see Figure 2, event B).

##### 2.2.2.3 University of New Hampshire (UNH)

As the UNH collection system lacks a size-selective inlet, we designate these measurements as total suspended particulates (TSP). Filters (Millipore Fluoropore, 90 mm, 1 μm nominal pore size) were collected for 12-hour periods, roughly 7:00 to 19:00 and 19:00 to 7:00 the next day, at a flow rate of ∼90 standard liters per minute. During intensive sampling periods (Figure 2), the collection frequency was increased to five samples per day: 1:00-7:00, 7:00-13:00, 13:00-17:00, 17:00-21:00, 21:00-1:00 (next day).

GT and UW filters were shipped to GT for analysis at below 0°C and subsequently stored frozen (-15°C), which is often a higher temperature than ambient temperature at time of sampling. UNH filters were analyzed in the field, soon after collection using the UNH IC system.

#### 2.2.3 Size-Resolved Particle Collection with a Micro-Orifice Uniform Deposit Impactor (MOUDI)

We collected a series of size-segregated samples with two non-rotating cascade impactors that were deployed alternately.^37^ Nominal particle diameter cut sizes are given in Table S1. Samples were collected at the CTC site for sampling periods ranging from two to four days with seven to nine stages (excluding inlet) and the after-filter. Table S2 lists all the samples used in this analysis. One or two bottom stages were occasionally removed to achieve a nominal 30 L/min flow rate at the start of the sampling. Flow rates changed from the start to the end of the sample period. Ambient air concentrations were determined from the average of the flow rates measured at the start and end of the sampling period. The median relative change was nominally ±8%, but larger during extreme cold periods (see Table S2). Stages were cleaned between sampling periods by soaking in methanol. Teflon filters (Whatman, 47 mm diameter, 1 μm pore size) were used as impaction substrates and for the after-filter (Whatman, 37 mm diameter, 1 μm pore size). These filters were extracted and analyzed in the same way at GT as bulk filters, discussed next.

#### 2.2.4 Filter Extraction

The filter analysis process included both untreated IC analysis and treatment with H_2_O_2_, shown graphically in Figure S1. Whole GT PM_2.5_ and all MOUDI samples were extracted into 10 mL Milli-Q water (Barnstead GenPure Pro >18 Mohm, pH 6.5). For UW size resolved high volume samples, one tenth of the upper rectangular impaction strips were used, and for the other size bins and backup filter (Dp<0.7 μm) 5.06 cm^2^ (2.54 cm diameter punch) was used for extraction. All were extracted into 15 mL Milli-Q water. Extracts were subsequently vortex-mixed for 5 seconds and sonicated for 30 min, then filtered (0.45 μm pore size polypropylene syringe filters) in preparation for IC analysis. Whole UNH filters were wetted with 0.5 mL spectroscopic grade methanol to improve particle extraction and then extracted into 20 mL of Milli-Q water. All UNH samples went through IC characterization either immediately after extraction or up to 4 h afterwards. The remaining extracts were stored at -20°C.

#### 2.2.5 Ion Chromatography Analytical Methods

Georgia Tech (GT) and the University of New Hampshire (UNH) performed independent IC analysis of various collected samples. At GT, the following method was used for the online PILS-IC and offline analyses of GT PM_2.5_, UW Hi-Volume filters, and all MOUDI samples: Anions were measured with a Metrohm 761 (Metrohm USA, Riverside, FL) IC unit with conductivity detection. Isocratic separation was achieved with a Metrosep A Supp-5 150/4.0 anion column at a flow rate of 0.7 mL/min and 10.5 MPa pressure with an 3.2 mM sodium carbonate and 1 mM sodium bicarbonate eluent at pH of 10.5. A suppressor with 4 mM sulfuric acid followed by a DI water rinse regeneration was used. A sample of 250 μL was injected onto the column from a sample loop. Minor overlap in the S(IV) and S(VI) peaks is estimated to result in less than 20% error in S(IV) concentrations. An example chromatogram is given in Campbell et al.^29^

For the UNH samples, anions were analyzed using a Dionex AS-11 column with a basic eluent of 10 mM NaOH at a flow rate of 1.0 mL/min. An ASRS suppressor was used in the recycle configuration. Sample was loaded onto the column from a 250 μL sample loop and good separation of S(IV) and S(VI) peaks with no overlap was achieved with this system. The approximate retention times of anions for the GT and UNH methods are listed in Table S3.

#### 2.2.6 Hydrogen Peroxide Treatment (GT and UNH)

Experiments show that for both the GT and UNH IC systems, HMS, and other S(IV) species co-elute. To isolate HMS, we treated the filter extractions with a solution of H_2_O_2_ just prior to IC analysis to convert free S(IV) to S(VI) with minimal effect on HMS. At GT, the treatment was set up as a continuous flow injection system with two reservoirs, one for the filter extract and one for a 6 mg/L H_2_O_2_ solution. A multichannel peristaltic pump merged the two at a ratio of five parts extract to one part 6 mg H_2_O_2_/L solution, which then passed through super-serpentine reactors (Global FIA, Fox Island, OR) with an approximate residence time of 7 min. Following the reactor, the product passed through the anion 250 μL sample loop and was immediately analyzed by ion chromatography. The UNH H_2_O_2_ treatment was done manually; 10 μL of 3% H_2_O_2_ was added to a 5 mL aliquot of the UNH filter extract. This solution was shaken briefly and allowed to sit for 10 min before IC analysis.

#### 2.2.7 Calibrations for S(IV), HMS, and Inorganic S(IV)

In GT, UW, and UNH analyses, two IC measurements were made for each filter extract: (1) the unaltered extract for standard anions, including S(IV), and (2) the extract treated with H_2_O_2_ for S(IV) speciation. The treatment is intended to isolate HMS from all other S(IV) species. HMS, as defined here, will include any other S(IV) species that is unreactive to H_2_O_2_. The S(IV) lost after H_2_O_2_ treatment are referred to as “other S(IV)” since it is determined by subtraction of HMS from S(IV). We incorporated a dilution correction factor for the addition of H_2_O_2_ (1.2 for GT and 1.002 for UNH) to the remaining S(IV) peak and calibrated it as HMS (i.e., used NaHMS in solution as a standard). For quantifying the other S(IV), we calibrated with HSO_3_^–^ using a stock solution of NaHSO_3_ but were unable to adequately calibrate for SO_3_^2–^, as diluted samples quickly convert to SO_4_^2–^. Assuming the other S(IV) reported contains a mix of HSO_3_^–^ and SO_3_^2–^, we expect slightly different IC conductivity responses from each species (estimated at approximately 12.5%), but that HSO_3_^–^ is a satisfactory approximation since we expect conditions in the particle and extract to favor HSO_3_^–^ over SO_3_^2–^ in the S(IV) equilibrium. In the online PILS-IC measurements, however, all S(IV) was calibrated as HMS which may overestimate the actual S(IV) concentrations by up to 4.7%, based on the difference between the HMS and HSO_3_^–^ calibrations and the average abundance of HMS we measured for PM_2.5_ S(IV) (70% of S(IV) is HMS).

#### 2.2.8 Measurements of Aldehydes

Gas phase aldehydes that could form adducts with S(IV) were also measured. Formaldehyde was measured by two instruments that were operational largely during different periods of the study; the NASA Goddard COmpact Formaldehyde FluorescencE Experiment (COFFEE)) instrument,^38,39^ which uses a laser-induced fluorescence technique, and a commercial AERIS Ultra^40^ instrument, which employs infrared absorption spectroscopy. These shared a PFA sample line, with a combined sample flow of ∼3 sLm. Good agreement was found between these measurements during periods of overlapping measurements and so the two measurements were combined to give a near continuous HCHO data set. Mixing ratios of higher molecular weight aldehydes were measured by proton transfer reaction time-of-flight mass spectrometry (PTR-ToF-MS) using a PTR-ToF-MS 6000 X2, (Ionicon Analytik GmbH, Austria). Raw PTR-ToF-MS mass spectra were processed with the commercial Ionicon Data Analyzer (IDA) software to identify the individual high-resolution peaks and extract their time series. The peaks were assigned the most likely elemental formula matching their exact mass within the experimental mass accuracy tolerance of +-10 ppm. When the VOC assignment is unambiguous, the relative uncertainty on the concentration derived from the propagation of the relative uncertainties on the transmission and the proton reaction rate constants is estimated to be within ±30%.^41^ For some aldehydes identified by mass to charge ratios, contributions from other species may occur, which is considered in the discussion below.

## 3 Results and Discussion

### 3.1 Evaluation of Method for Speciation of S(IV) by Isolating HMS with Hydrogen Peroxide

HMS may undergo conversion to SO_4_^2–^ between filter extraction and IC analysis. Although HCHO can be added to stabilize HMS, Campbell et al.^29^ showed that without adding HCHO to preserve the inorganic S(IV) for extended durations, only 2-5% of HMS mass is converted to SO_4_^2–^ through the GT analysis process. We repeated and expanded upon these tests to characterize the stability of HMS, HSO_3_^–^, and SO_3_^2–^ across various steps of the extraction, IC analysis, and H_2_O_2_ treatment for S(IV) speciation. Different degrees of testing were performed by the GT and UNH groups.

#### 3.1.1 HMS Stability and Reaction with Hydrogen Peroxide

At GT, benchtop experiments on the stability of HMS were performed using NaHMS in Milli-Q water. With a fresh (prepared the same day) solution of NaHMS, less than 2% of HMS mass was oxidized to SO_4_^2–^ through solution preparation and passing through the column to the detector in the basic carbonate/bicarbonate mobile phase (eluent) (see Table S4 column 2), in agreement with Campbell et al. (2022).^29^ This value increased to about 3% for an elapsed time of about 24 h, (see Table S4 column 3). We view this as the upper bound for the error on filter HMS lost during sample preparation and analysis by both GT and UNH methods, assuming HMS in ambient aerosols behaves the same way as the HMS ion from the NaHMS salt.

Previous studies report HMS is resistant to oxidation by H_2_O_2_,^21^ but we also tested this for the conditions of the GT H_2_O_2_ speciation system. We found that approximately 10 to 16% of measured HMS mass is lost to the reaction of the standard (NaHMS) with H_2_O_2_, where higher standard concentrations correspond to a lower fraction of mass lost (see Table S4 columns 4 and 5), possibly due to a small portion of the HMS adduct dissociating into inorganic S(IV) (and HCHO), which then reacts with H_2_O_2_. This leads to a possible underestimation of HMS by 10 to 15%, which is not accounted for in the following analysis and viewed as a measurement uncertainty. We also found no evidence for conversion of ambient HMS to SO_4_^2–^ by comparing results from the various filter methods, which have different HMS concentrations in the extracts (Figure S2).

#### 3.1.2 Bisulfite Stability and Reaction with Hydrogen Peroxide

We performed experiments to test the stability during IC analysis and removal efficiency of HSO_3_^–^ via oxidation with H_2_O_2_. Bisulfite (HSO_3_^–^) is the dominant S(IV) species in the SO_2(aq)_ - HSO_3_^–^ - SO_3_^2–^ equilibrium in the ambient aerosol pH range^3^ and one of two inorganic S(IV) species we wish to isolate HMS from. HSO_3_^–^ undergoes some conversion to SO_4_^2–^ in the basic eluent, but to a greater degree than HMS. We find that HSO_3_^–^ losses can range from 11 to 14% for initial concentrations between 0.25 to 2 mg/L (Table S5).

The HSO_3_^–^ standard was found to be stable in the extract solution, and this stability is enhanced upon refrigeration. Based on measuring the S(IV) (HSO_3_^–^) peak of a 2 mg/L solution at differing times (Table S6), we expect about 5% error in the measurement of HSO_3_^–^ in filter samples over the sample analysis period. Incorporating results from conversion in the eluent discussed above (Table S5) and the time evolution of the S(IV) peak (Table S6) we estimate a combined error in our measurement of other S(IV) by up to approximately 15%. Bisulfite is efficiently removed by the H_2_O_2_ treatment method (Table S5), although the removal efficiency decreases as the HSO_3_^–^ concentration decreases, likely due to the slower kinetics from the dilution of available reactive species. In most samples, we expect up to 5-7% error in our estimate of other S(IV) attributed to uncertainties in HSO_3_^–^.

#### 3.1.3 Sulfite Stability and Reaction with Hydrogen Peroxide

Attempting to measure sulfite (SO_3_^2–^) with the IC can be challenging because it is unstable in solution at low concentrations, rapidly converting to SO_4_^2–^. For solutions containing only SO_3_^2–^, we observed the S(IV) peak rapidly decay and the S(VI) peak grow in successive IC measurements over the period of extraction and characterization, reaching 33% loss over a period of approximately 4.5 h (Table S7). This loss increases overnight; for 25-750 μg/L SO_3_^2–^ standards to 81-96%. Our results show that H_2_O_2_ also removes SO_3_^2–^ (see Table S8). UNH found 89% loss of SO_3_^2–^ after 10 min, with marginal additional loss out to 1 h. This is the basis for the UNH H_2_O_2_ treatment method discussed above.

Overall, we found that HMS and HSO_3_^–^ are stable once extracted from the filter into water, but SO_3_^2–^ is not. We did not test the stability of S(IV) and HMS on filters when stored at -15°C for long periods, but below we compare GT and UNH filters that involved different storage times. In tests of the S(IV) speciation analysis method, we found that HMS is resistant to oxidation by H_2_O_2_ while HSO_3_^–^ is almost completely depleted under the same conditions. Sulfite is also significantly removed by H_2_O_2_, but it is also unstable over the extraction and analysis period and quickly converts to SO_4_^2–^. As reaction with SO_3_^2–^ is the primary route of HMS formation, we believe that HMS is the only species remaining after peroxide treatment, assuming that there are no other additional S(IV) species present in the samples that are resistant to the H_2_O_2_ treatment.

### 3.2 Comparison of Methods for Measuring Sulfur Species

**Figure 1:**
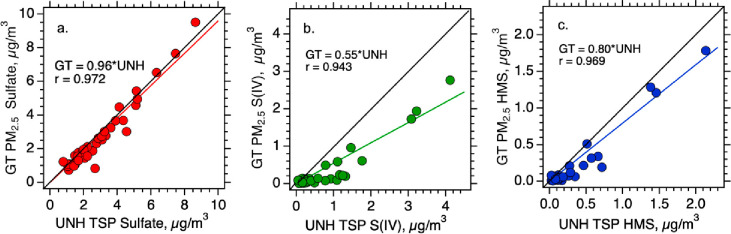
Comparison of filter collection with IC analysis by UNH and GT. UNH collected TSP and analyzed the filters in the field, GT measured PM_2.5_ and analyzed the filters after 4 to 8 weeks of storage. Slope of 1 line (black) and orthogonal regression results are shown with the intercept forced through zero.

Two independent measurements and analyses of S(IV), HMS and SO_4_^2–^ by the UNH and GT groups, allow comparison of experimental methods that can be affected by differences in sampling upper particle diameter size cutoffs and stability of S(IV) species on filters in the 1 to 2 months before GT analysis. Figure 1 shows a comparison of SO_4_^2–^, S(IV) and HMS. Sulfate does not appear to be prone to sampling artifacts; the excellent agreement (slope of 0.96) demonstrates no biases in sampling and analytical methods between the UNH TSP and GT PM_2.5_ and that there is little SO_4_^2–^ in the coarse mode. For S(IV) and HMS, however, GT PM_2.5_ amounts to 55% and 80% of the UNH TSP, respectively. (A similar analysis for other S(IV) gives a fraction of 40%). These values mean that either other S(IV), and HMS to a lesser extent, are more prone to evaporation from filters (i.e., GT samples were measured after an extended period of storage while UNH samples were stored frozen for less than 18 h before extraction and analysis), or that there are significant amounts of S(IV) in the coarse mode, or both. More detailed size resolved data discussed in Section 3.5 show that some of these differences are due to contributions from the coarse mode included in the UNH TSP measurement but not in the GT PM_2.5_ measurement. Also, similar differences in TSP and PM_2.5_ HMS and other S(IV) were observed on a subset of GT filters (four filters) analyzed by UNH in the field, which would not be prone to artifacts associated with extended PM_2.5_ filter storage prior to analysis suggesting the contrasts are mostly due to the differences in the size of particles included, I.e., PM_2.5_ and TSP.

### 3.3 Sulfate, S(IV), and HMS Time Series

Previous measurements of species concentrations in Fairbanks winters described by Campbell et al.^29^ during 2020 and 2021 were of relatively short duration (in part due to restrictions imposed by COVID) and served as exploratory work prior to the main ALPACA intensive study, reported here. Concentrations of various key species during the three winters were comparable, including during cold events when S(IV) and SO_4_^2–^ were both significantly higher than the study average (see Figure S3 and Table S9. The consistency between three winters indicates that the following results from the ALPACA intensive are likely common in Fairbanks winters.

**Figure 2:**
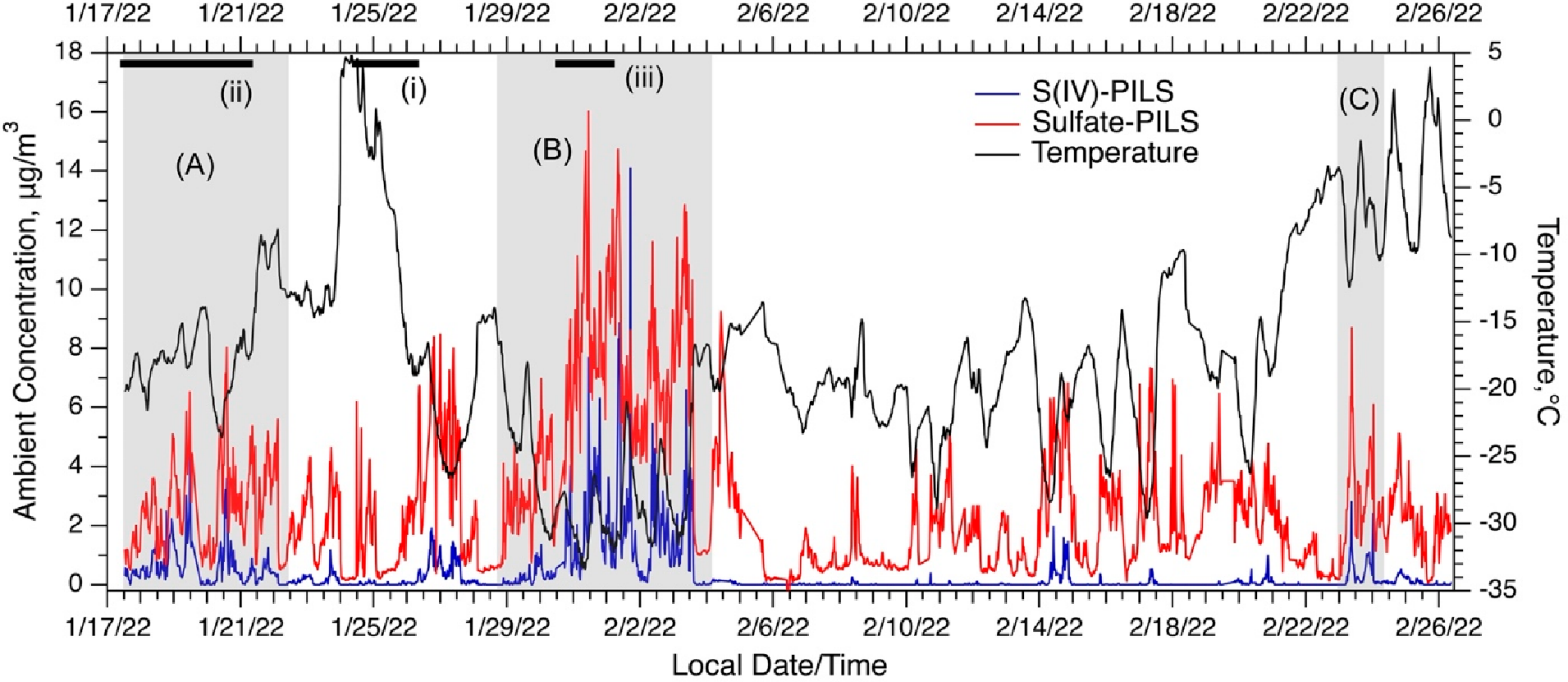
PILS online measurements of PM_2.5_ sulfate and S(IV) for the ALPACA intensive study. Temperature is also shown along with periods used in contrasting case studies shaded in gray. Black bars indicate MOUDI sampling periods to characterize particle size distributions that are plotted in Figure 5.

PM_2.5_ measurements of S(IV) and SO_4_^2–^ from ALPACA, along with ambient temperature, are shown in Figure 2. We define key events and periods of interest in Figure 2. In chronological order, these periods are (A) moderate levels of S(IV) at the start of the ALPACA campaign, (B) a pollution event of high PM_2.5_ mass concentration during an extreme cold period where temperatures dropped to near -35°C (referred to as the cold event), and (C) an end-of-study S(IV) event during a period of higher temperatures.^32^

**Figure 3:**
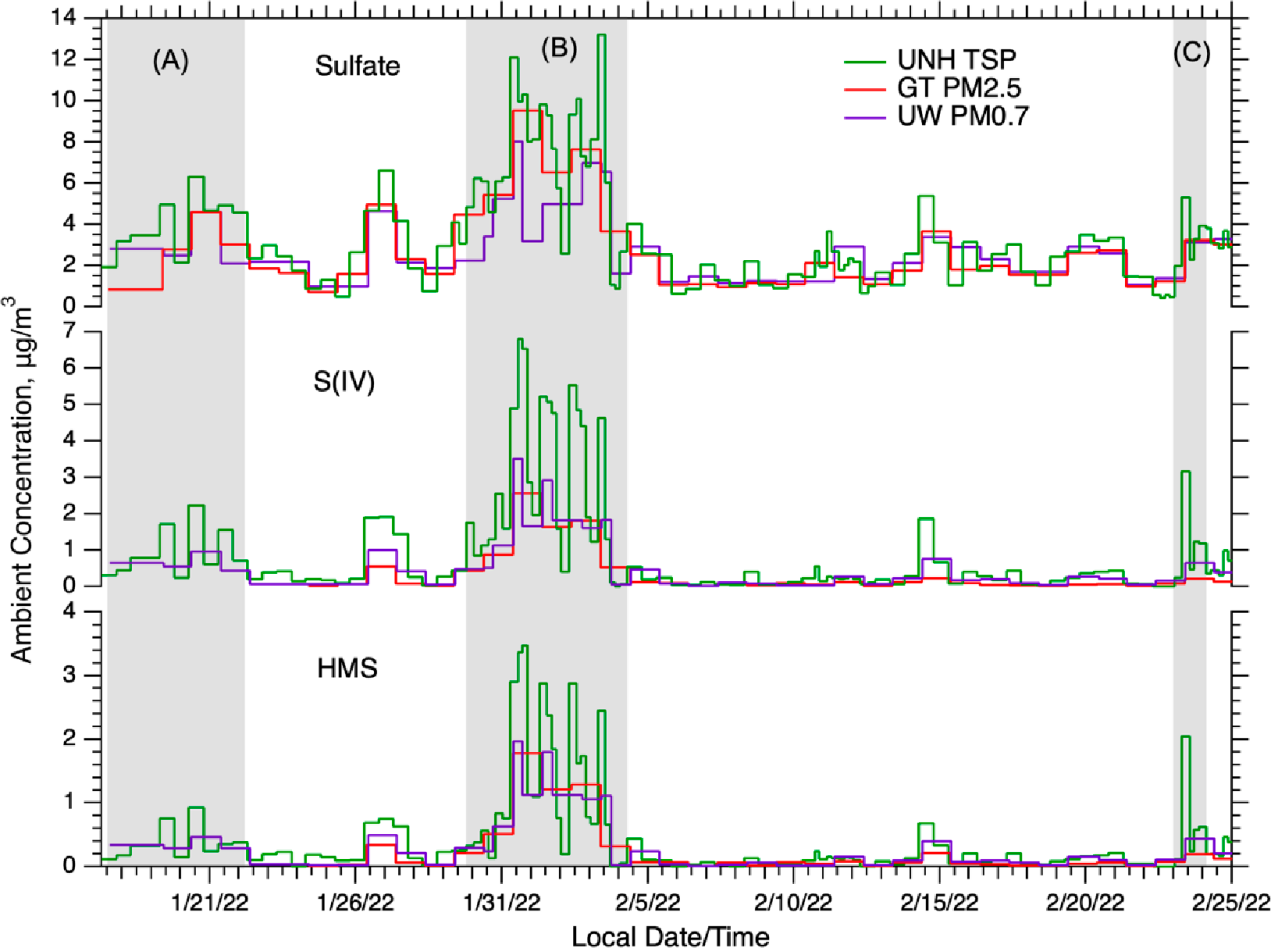
Time series of sulfate, S(IV) and HMS determined by three different filter sampling systems. Gray shaded areas are periods analyzed in more detail.

Although we did not use the online PILS measurement system to speciate S(IV) (Figure 2), it is possible to generate a lower time resolution time series of HMS, S(IV) and SO_4_^2–^ with some degree of particle size resolution based on the multiple filter samples collected by the three research groups. Figure 3 shows the UNH (TSP), GT (PM_2.5_) and UW final filter stage (PM_0.7_) for SO_4_^2–^, S(IV) and HMS. Generally, SO_4_^2–^ concentrations were similar within the three filter data sets, implying that it is mainly found in PM_0.7_, consistent with primary emissions from combustion sources tending to produce small particles and isotopic results that find the majority of SO_4_^2–^ in downtown Fairbanks was primary (62 ± 12% by mass of PM_2.5_).^36^ In contrast to SO_4_^2–^, there is a large amount of S(IV), and to a lesser extent HMS, in the coarse mode (i.e., TSP > PM_2.5_ and PM_0.7_), which is consistent with Figure 1.

### 3.4 Fraction of HMS in S(IV)

**Figure 4:**
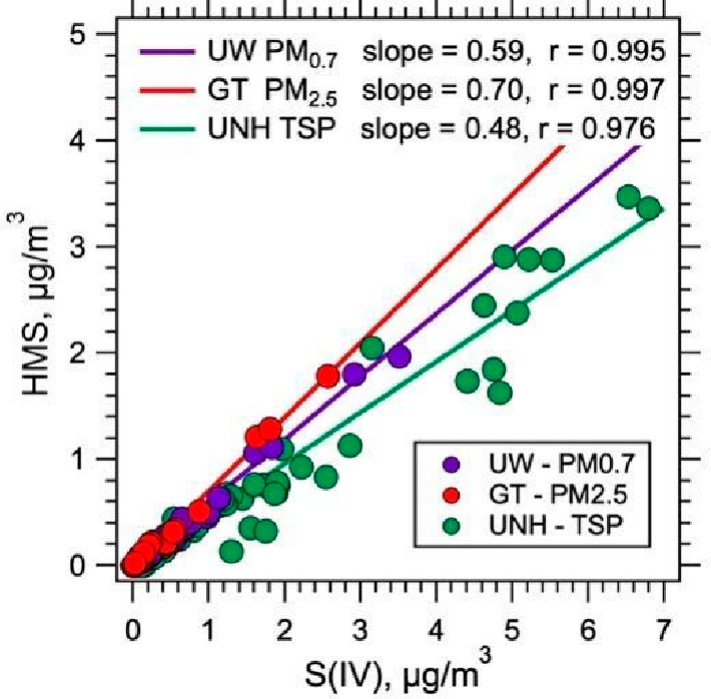
HMS versus S(IV) from filter samples that collected particles over three different size ranges. The slopes are orthogonal regressions and give the HMS fraction of S(IV).

To quantify the fraction of HMS in S(IV), we performed a linear regression on the concentrations of HMS obtained from the H_2_O_2_ treatment to the overall measured S(IV) using filters from each particle size bracket. Figure 4 shows that the fraction of HMS in S(IV) is 0.59, 0.70, and 0.48, for PM_0.7_, PM_2.5_, and TSP, respectively, roughly comparable to the values previously reported for smaller cloud droplets by Rao et al.^21^ The maximum in the middle size bracket indicates that relative to all S(IV) species, a larger proportion of HMS is between particle diameters of 0.7 and 2.5 μm and there is a high degree of linearity between HMS and S(IV); Pearson’s correlation coefficients range between 0.97 to 0.99. One possible reason for both observations is that HMS and S(IV) are formed by a similar process. Since HMS is formed exclusively through aqueous-phase chemistry, this suggests that S(IV) is also predominantly formed through aqueous-phase chemistry (which is discussed more below). A common source is particle water uptake of SO_2_, the gaseous starting reagent for inorganic S(IV) and one precursor of HMS. The higher scatter in TSP suggests that this linkage is weaker for coarse mode particles.

Some cause for correlation is also related to meteorology and that HMS is a significant component of S(IV). Direct comparisons of HMS and other S(IV) concentrations (Figure S4a), (other S(IV)= S(IV) - HMS,) show that for PM_2.5_, the ratio of HMS to other S(IV) is 2.32 and the two are highly correlated (Pearson’s correlation r^2^=0.963), whereas for TSP the ratio is 0.911 and r^2^=0.911. The regression results comparing HMS/SO_4_^2–^ and other S(IV)/SO_4_^2–^ (Figure S4b) give similar results, but with more scatter. This implies that for fine particles (PM_2.5_), concentrations of HMS are a little over double those of other S(IV) and the two are possibly coupled in some manner. Including the coarse mode data (i.e., TSP), however, shifts the slope to less than one (0.91), suggesting that most other S(IV) is in the coarse mode, and it may be associated with a different source than the fine mode other S(IV).

### 3.5 Size Distribution of Sulfate, S(IV), HMS, and Inorganic S(IV)

**Figure 5:**
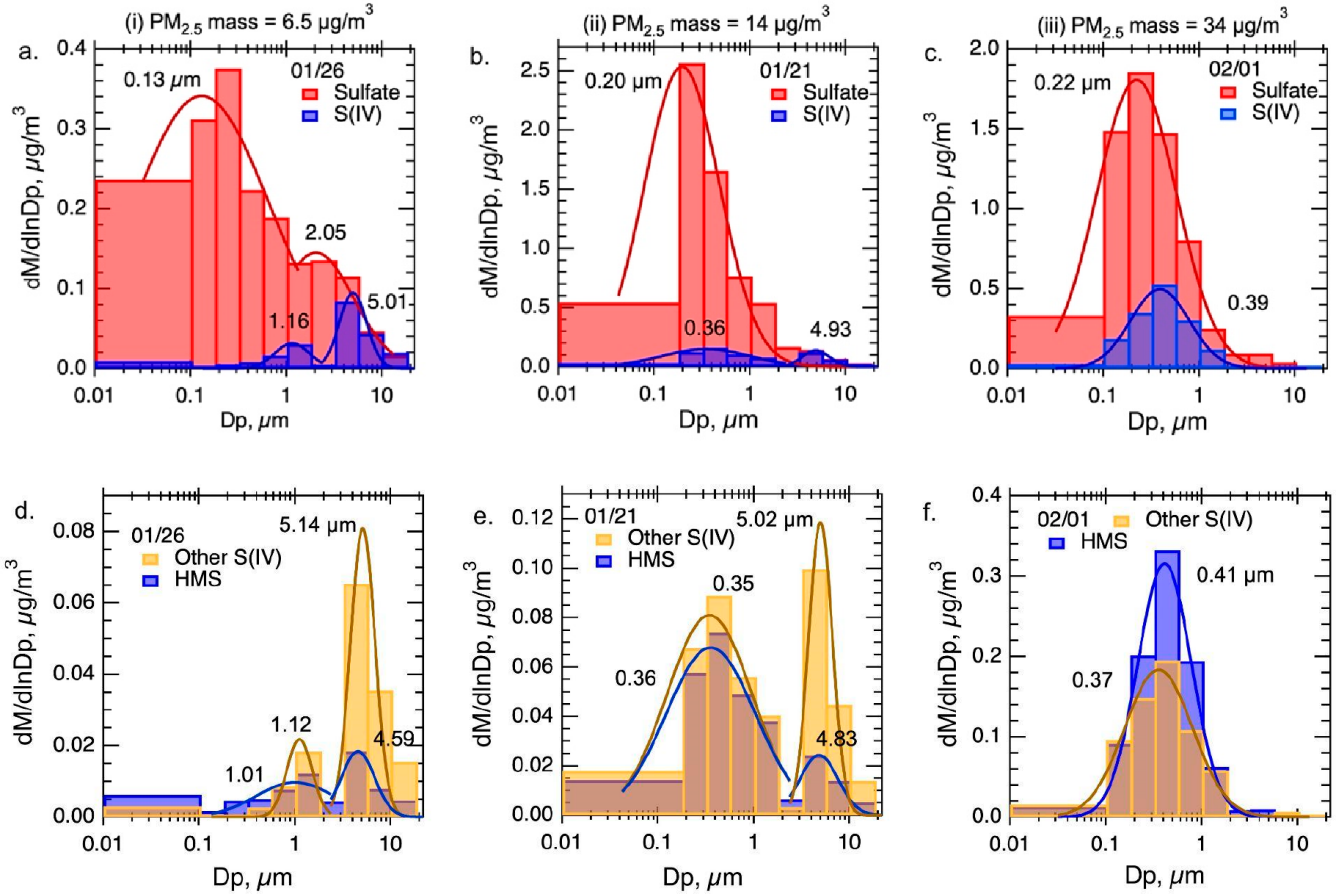
Selected size distributions from MOUDI measurements during the ALPCA study for sample ending dates shown (month/day; 01/26, 01/21 and 02/01). These sampling periods are shown in Figure 2 and correspond to periods of (i) low, (iii) moderate and (iii) high PM_2.5_ mass concentration, given at the top of each column of plots. All species are plotted to zero (not stacked). The smallest size channel is from the MOUDI after filter with lower range arbitrarily set at 0.01 μm aerodynamic diameter. Three MOUDI measurements (each column is one MOUDI measurement) were selected for a range of PM_2.5_ and S(IV) concentrations; (a) (d) relatively low, (b) (e) moderate and (c) (f) high. The lognormal fits for various modes and corresponding geometric mean aerodynamic diameters are also shown. More fit details, including geometric standard deviation and the uncertainty in fit parameters are given in Figure S6. Other S(IV) is equal to the difference in S(IV) and HMS.

MOUDI sampling occurred over nominally 2 days and so lacks time resolution but provides better particle size resolution than comparison between bulk filter samples. Figure 5 shows select size distributions of SO_4_^2–^, S(IV), HMS, and other S(IV) over periods of low (10:00 Jan 24 to 9:15 Jan 26), moderate (11:00 Jan 17 to 9:00 Jan 21) and high PM_2.5_ concentrations (10:00 Jan 30 to 9:00 Feb. 1). Sampling periods are identified (as bars) in Figure 2 and detonated as (i) low, (ii) moderate and (iii) high PM_2.5_ mass periods, respectively. Average PM_2.5_ mass concentrations during the low (i), moderate (ii), and high (iii) sampling periods were 6.5, 14, and 34 μg/m^3^, respectively. The complete collection of MOUDI size distributions of SO_4_^2–^ and S(IV), and HMS and other S(IV) are shown in Figure S5 and the masses of these species in the fine and coarse modes are given in Table S10.

The MOUDI results show contrasts in size distributions between the different sulfur species. Sulfate is largely found in the fine mode (D_p_ < 2.5 μm). Sulfate geometric mean diameter (D_pg_) and geometric standard deviation (σ_g_) for the moderate (ii) and high (iii) PM_2.5_ mass concentration periods (Figure 5b and 5c) are roughly similar, with D_pg_ = 0.2 μm and σ_g_ = 2.5 (see Supplemental Figure S6 for more details on the fit data, including σ_g_). For the low PM_2.5_ mass concentration (i) period (Figure 5a), SO_4_^2–^ is shifted to slightly smaller sizes; D_pg_ was 0.13 μm and the distribution was broad (σ_g_ = 5.1). Shift in the SO_4_^2–^ distribution (D_pg_) going from high (iii) to low (i) PM_2.5_ mass concentration periods is consistent with SO_4_^2–^ being a combination of smaller primary particle emissions (mainly from residential fuel oil heating) and slightly larger particles from the added secondary SO_4_^2–^ formed in the aqueous phase of PM_2.5_ (e.g., aqueous reactions of SO_2_ with NO_2_, O_3_ or H_2_O_2_).^36^ Coarse mode SO_4_^2–^ may also be from a different source, such as local thermal generating units burning coal^42^ that emit from stacks above the strong temperature inversion. This is consistent with greater influence from this source detected by surface monitors during clean periods when the inversion is weaker and there is greater vertical mixing and dispersion, resulting in lower overall PM_2.5_ concentrations and proportionally higher SO_4_^2–^ concentrations in coarse to fine modes (Figure 5a) relative to the moderately and high PM_2.5_ mass concentration periods (Figure 5b, 5c) when the inversion limits their impact. Alternatively, cleaner conditions may represent the background air quality in general, which includes aged SO_4_^2–^ from long range transport and associated with Arctic haze.^43,44^

S(IV) is also shown in Figures 5a, 5b and 5c and has generally much lower concentrations than SO_4_^2–^, except in the coarse mode in the low (i) and moderate (ii) PM_2.5_ mass concentration cases and tends to be in differing size ranges than SO_4_^2–^. The D_pg_ of fine mode S(IV) (0.36 to 0.39 μm for moderately (ii) and high (iii) PM_2.5_ mass concentration periods) are higher than SO_4_^2–^ (∼0.2 μm), pointing to differences in sources, such as mainly aqueous chemistry for production of S(IV), instead of a significant contribution of primary emissions smaller fine mode SO_4_^2–^. These differences in sizes show that a fraction of the SO_4_^2–^ (e.g., primary SO_4_^2–^) is externally mixed with S(IV). During the cleaner period ((i) Figure 5a), fine mode S(IV) shifted to larger sizes, (D_pg_ of 1.16 μm), which Figure 5d shows is largely due to high contributions of other S(IV).

The corresponding HMS and other S(IV) size resolved data for the three periods are shown in Figures 5d, 5e, and 5f. In the low (i) and moderate (ii) PM_2.5_ mass concentration cases, the coarse mode other S(IV) concentration is high relative to the fine mode. It was noted above that coarse mode S(IV) may have a different source, e.g., coal-fired thermal generating units. These data indicate it is largely composed of other S(IV). S(IV) has been reported in past studies to be emitted from coal-burning associated with fly ash.^45^ Moving from low (i) to moderately polluted cases (ii), HMS becomes more prominent in the fine mode, and in the high (iii) PM_2.5_ mass concentration period (cold event Figure 5f) HMS dominates over other S(IV), likely reflecting the increasing extent of its specific aqueous chemistry. For moderate (ii) and high (iii) PM_2.5_ mass concentration conditions, the D_pg_ of HMS and other S(IV) is nearly the same and the two distributions are very similar. Because these compounds are found in similar size ranges, both could be mainly formed through an aqueous process.

There is some inconsistency between the MOUDI data and contrasts between filter GT PM_2.5_ and TSP. The large differences between UNH TSP and GT PM_2.5_ seen in Figures 1b and 3 for S(IV), and especially for other S(IV), are not seen in the MOUDI data as a significant coarse mode. This could be attributed to any of the following: higher time averaging in MOUDI data that mixes periods of high and low S(IV) concentrations, the MOUDI excluding larger particles measured by the filter TSP method, partial loss of S(IV), mainly other S(IV), due to the delay in the MOUDI and GT PM_2.5_ filter analyses compared to the UNH TSP analysis, or some combination of all.

### 3.6 Effect of Temperature and Relative Humidity on S(IV) Speciation

In the following we contrast the response of S(IV) and speciated S(IV) (HMS and other S(IV)) to temperature and RH, factors critical to aqueous aerosol formation, to further assess if they have a common formation process.

#### 3.6.1 Temperature

**Figure 6:**
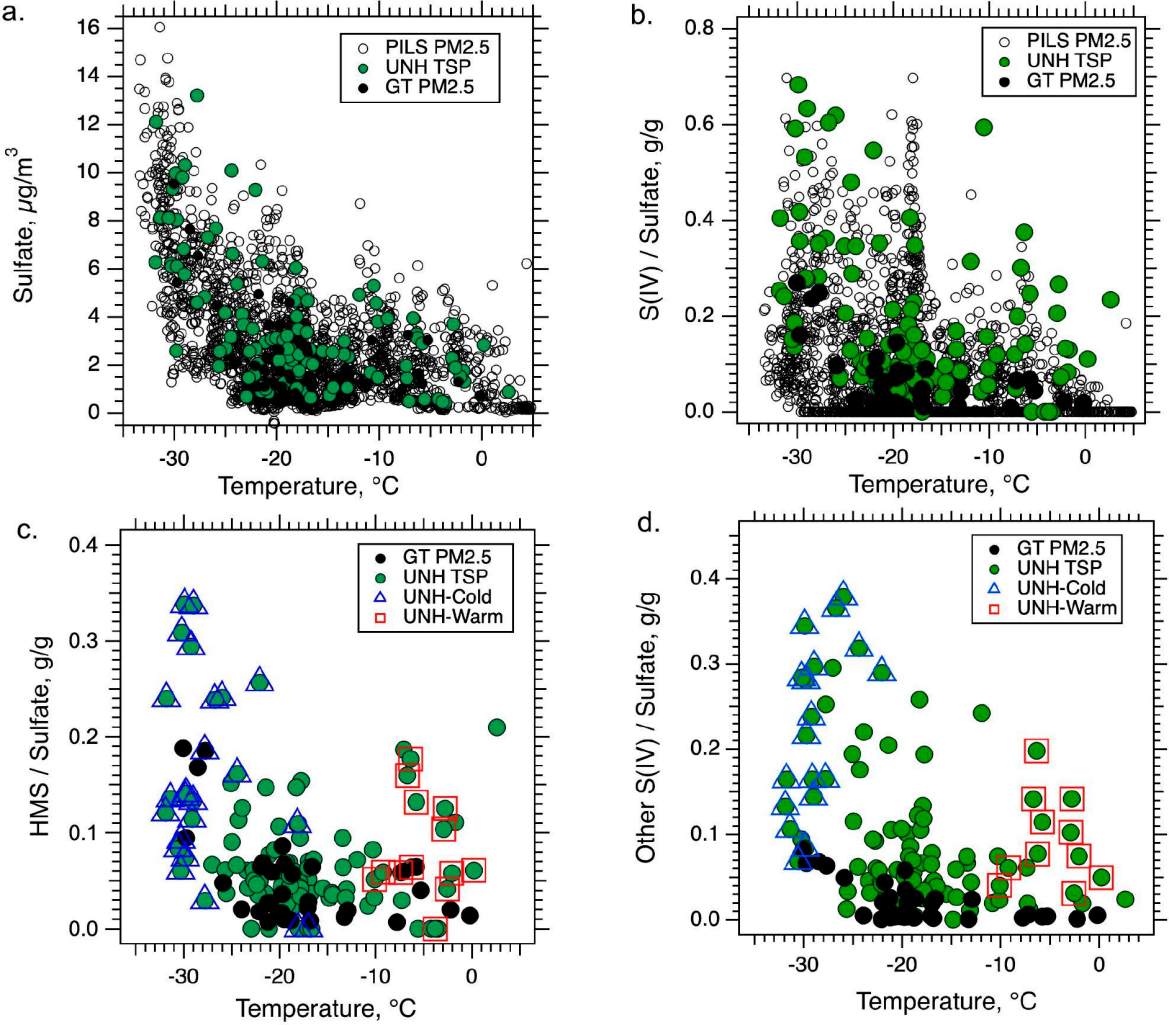
Variation of sulfur species with surface temperature at the CTC site based on various measurement methods. (a) Sulfate concentration measured online with the PILS and with UNH TSP filters and GT PM_2.5_ filters. (b) Ratio of S(IV) to sulfate, (c) ratio of HMS to sulfate and (d) ratio of other S(IV) to sulfate. The PILS did not speciate S(IV) with the H_2_O_2_ treatment so only filter data are shown in plots (c) and (d), which also shows the UNH data collected during the cold ((B) in Fig. 2) and warm ((C) in Fig. 2) events. Other S(IV) is equal to the difference in S(IV) and HMS.

Cold events in Fairbanks are associated with pollution episodes of high PM_2.5_ mass concentration in Fairbanks. Strong temperature inversions limit dispersion,^31^ coincident with increased emissions from greater residential heating with wood and fuel oil burning which contain sulfur species that produce gas phase SO_2_ and SO_4_^2–^ aerosol. This in large part accounts for the increasing concentration of SO_4_^2–^ with lower temperatures shown in Figure 6a.

The ratio of S(IV) to SO_4_^2–^ also increases as temperature decreases, meaning that although SO_4_^2–^ increases with lower temperature (Figure 6a), S(IV) increases at an even higher rate, possibly exponential (Figure 6b). Campbell et al.^29^ showed a similar result for the winters of 2020 and 2021 and attributed this to the increased solubility (higher Henry’s Law constants, or lower vapor pressures) of the HMS precursors, HCHO and SO_2_, with lower temperature., The resulting higher concentrations of HCHO, HSO_4_^–^ (R2) and SO_4_^2–^ (R3) in particle water would lead to greater HMS production, but also possibly enhance secondary SO_4_^2–^ production through other aqueous phase routes (e.g., reactions of S(IV) with H_2_O_2_, or NO_2_^36^). There is no primary HMS but both primary and secondary SO_4_^2–^,^36^ so depending on the proportion of secondary SO_4_^2–^ relative to primary SO_4_^2–^ as a function of temperature, normalizing by SO_4_^2–^ may underestimate the increase in S(IV) species with lower temperature since most secondary SO_4_^2–^ is also expected to be formed through an aqueous phase process.^36^ In any case, there is a clear substantial enhancement in S(IV) with lower temperature which is likely driven by the increased uptake of precursor species to the aqueous particles as temperature drops and the particles remain super-cooled water.

Figure 6c and 6d shows that HMS/SO_4_^2–^ and other S(IV)/SO_4_^2–^ have similar trends with temperature. A noteworthy feature in both plots is data collected during the warm event ((C) in Figures 2 and 3). For these data, HMS/SO_4_^2–^ and other S(IV)/SO_4_^2–^ do not follow the broader trend with temperature. The warm event was associated with unusually high RH, suggesting that higher liquid water levels promoted aqueous reactions despite the warmer conditions by providing more volume for the uptake of the S(IV) precursors and the aqueous-phase reactions. Overall, the similar trends of HMS/SO_4_^2–^ and other S(IV)/SO_4_^2–^ again point to a similar formation process.

#### 3.6.2 Relative Humidity and Particle Liquid Water

**Figure 7:**
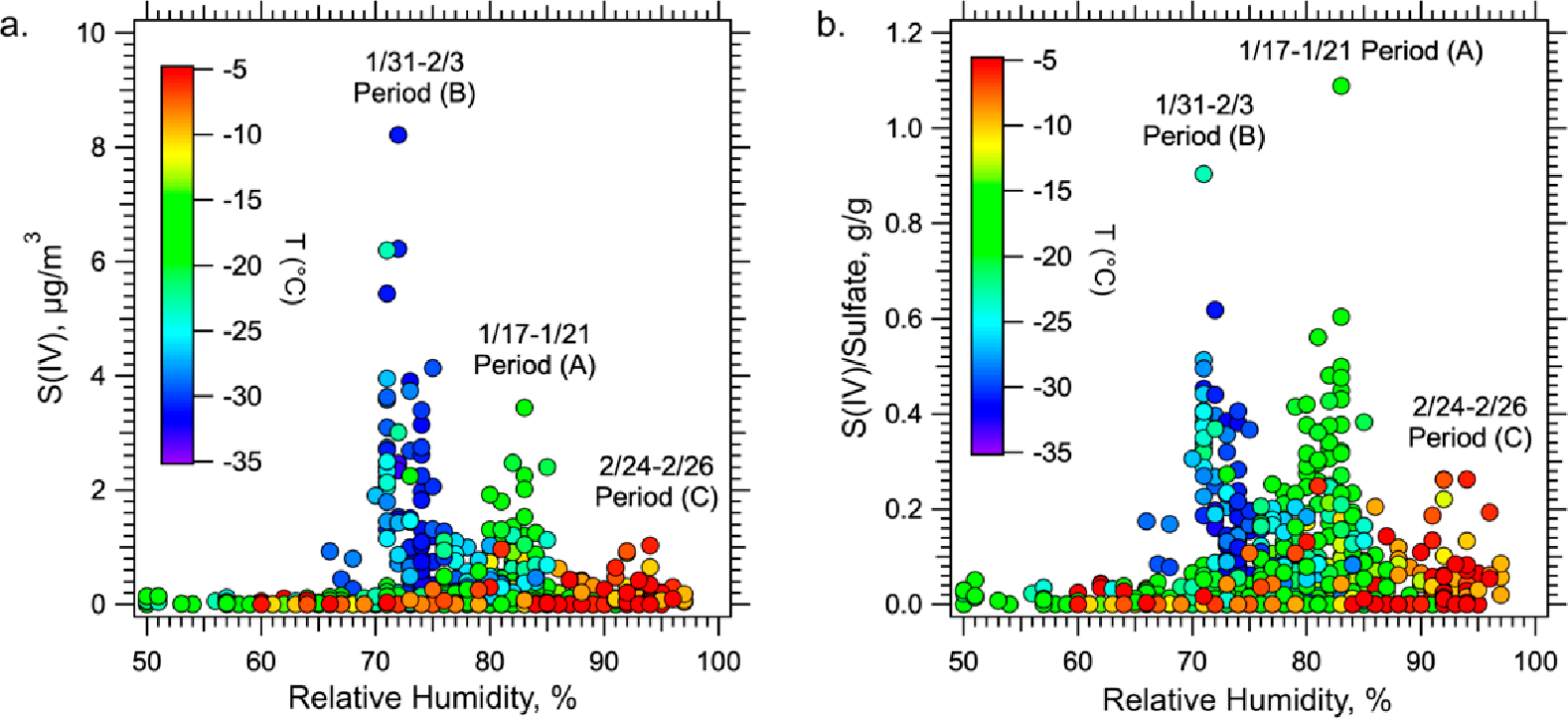
PM_2.5_ S(IV) concentrations as a function of ambient RH for all data measured during ALPACA with the PILS. Data are colored by ambient temperature and the various events of higher concentrations identified in Figure 1 are labeled as periods (A), (B), and (C).

Assuming equilibrium between the particles and water vapor, aerosol liquid water content (ALWC) has a strongly nonlinear dependence on relative humidity; liquid water gradually increases with RH up to approximately 85 to 90%, after which point ALWC increases rapidly as saturation (100% RH) is approached (see Figure S7). The number of species in air that are taken up by particle water, (like SO_2_ with conversion to inorganic S(IV)), or that are formed exclusively in particle water, (like HMS) tend to scale with ALWC. The RH in this study was generally below 85%, except for the warm period (C) at the end of February. Figure 7a shows that S(IV) concentrations increase with lower RH, where the coldest events (B) had the lowest RH and yet highest S(IV) concentrations, opposite of what would occur if ALWC controlled S(IV) uptake.^24^Figure 7b shows S(IV)/SO_4_^2–^ to remove some of the concentrating effect of lower inversion heights with lower temperature (accounted for by primary SO_4_^2–^), and the strong dependence of water uptake on SO_4_^2–^ concentrations. Lower RH will reduce ALWC and lead to lower S(IV) concentrations in air, again opposite to what is observed. Hence, the overall increasing trends of S(IV) with lower temperature (Figure 7) indicates that the temperature effect on S(IV) concentrations (Figure 6) is larger than the effect of decreased ALWC for the cold conditions of this study. The weaker influence of ALWC is most clearly observed when contrasting the warm period at the end of the study (C) to periods (A) and (B). During the warm period, the RH exceeded 90% and calculated ALWCs reached 80 μg/m^3^; the RH for the other periods was between 70-85% and ALWC was 30 to 40 μg/m^3^, yet much higher S(IV) concentrations were observed during these periods of lower liquid water concentrations due to much lower temperatures.

### 3.7 Other Aldehydes as a Source for Other S(IV)

Our analysis shows that HMS and other S(IV) (i.e., S(IV)-HMS) are closely linked during the more polluted cold events of high PM_2.5_ mass concentration. A possible reason is that a fraction of other S(IV) was formed in a similar way to HMS, for example, the aqueous reaction of other aldehydes with HSO_3_^–^ or SO_3_^2–^. Olson and Hoffmann^12,46^ identified a series of additional aldehydes that could form hydroxyalkylsulfonates (aldehyde-S(IV) adducts) and serve as S(IV) reservoirs in cloud water. These aldehyde-S(IV) species are more unstable than HMS and would be prone to dissociation during the extraction and analysis process of our analytical methods and be detected as other S(IV).^12^ Some of these gas phase aldehydes were measured in Fairbanks and found at lower concentrations than HCHO, but still significant. Table 1 lists HCHO and additional measured aldehydes and the adducts that could be formed. Based on their concentrations, adducts of acetaldehyde, glycolaldehyde and methylglyoxal could form aqueous phase HES, DHES and HAMS, respectively (see Table 1). The various rate constants of these reactions are expected to vary between species^47^ and are much smaller than HCHO forming HMS (R6) so the particle phase adduct concentrations are expected to be much lower than HMS and not necessarily in proportion to their gas-phase concentrations; although rate constants are uncertain at the extreme low temperatures and high ionic strengths of supercooled aqueous PM_2.5_ in Fairbanks.^48^ There is moderate (HCHO and acetaldehyde r = 0.729) correlation to low correlation (HCHO and glycolaldehyde r = 0.399) between these other aldehydes and HCHO suggesting different sources (see Figure S8). Note that low correlations could also be due to interferences from other species with same mass to charge ratios, see Table 1 and Table S11. However, the strong effect of extreme low temperature on HMS formation would likely also apply to these other adducts and account for the observed close linkage between HMS and other S(IV).

**Table 1:** Summary of Measured Aldehydes, Possible Aldehyde-S(IV) Adducts, and Observed Study-Average Aldehyde Concentrations

Aldehyde	Aldehyde-S(IV) Adduct	Adduct Formula	Aldehyde Concentration, ppbv Median ± Stdev
Formaldehyde	Hydroxymethanesulfonate (HMS)	CH2(OH)SO3-	2.57 ± 1.80
Acetaldehyde	I-Hydroxy-1-ethanesulfonate (HES)	CH3CH(OH)SO3-	1.46 ± 1.18^a^
Glycolaldehyde	1,2-Dihydroxy-l-ethanesulfonate (DHES)	CH2(OH)CH(OH)SO3-	1.39 ± 1.41^a^
Benzaldehyde	Hydroxyphenylmethanesulfonate (HPMS)	C6H5CH(OH)SO3-	0.020 ± 0.027^a^
Methylglyoxal	Hydroxyacetylmethanesulfonate (HAMS)	CH3COCH(OH)SO3-	0.12 ± 0.06^a^

a These concentrations correspond to the upper range as interferences from other VOCs on the PTR-ToF-MS protonated ion signals cannot be avoided.^51^ This is specially the case for signal at the corresponding protonated glycolaldehyde m/z that suffers strong interferences, mostly from acetic acid, in areas impacted by biomass burning emissions.^52,53^ See Table S11 for more details.

In addition to hydroxyalkylsulfonates, other chemical forms of S(IV) linked to aqueous particle SO_2_ uptake could show a similar behavior to HMS, such as metal-S(IV) complexes or just free HSO_3_^–^ and SO_3_^2–^. However, both are likely minor contributors to the other S(IV). The most prevalent PM_2.5_ metal measured during ALPACA was iron, with water-soluble iron <5 ng/m^3^^36^ (metal-S(IV) complex must be dissociated to be detected as S(IV) so water-soluble Fe is used), a factor over 30 times smaller than other S(IV) (WS Fe/other S(IV) = (5 ng/m^3^)/(160 ng/m^3^), see Table S5). Very low concentrations of free inorganic S(IV) are expected based on equilibrium predictions for typical atmospheric temperatures, however, the effects of extreme cold on the reactions R1, R2, and R3, (e.g., assumed equilibrium) is not clear.

### 3.8 Comparative Role of HCHO and SO_2_ in HMS Formation

Fairbanks and the NCP are the two regions where high concentrations of HMS have been recorded, both during cold periods (wintertime). Over the NCP, Moch et al.^17^ find HMS formation limited by HCHO since SO_2_ concentrations exceed HCHO by over an order of magnitude and HCHO loss by other processes were slow. They conclude that to improve air quality by lowering the contribution of HMS to PM_2.5_ mass concentration, control strategies should focus on transportation emissions, the source of HCHO.^17^ A similar scenario is found in Fairbanks, where SO_2_ concentrations are significantly higher than HCHO. The study average HCHO/SO_2_ ratio is 0.26 ppb/ppb and zero intercept regression slope is 0.21, (Figure S9). HCHO sources in Fairbanks are also largely from transportation,^49^ whereas SO_2_ is mainly from residential heating with fuel oil.^32^ A more detailed investigation on the relative roles of HCHO and SO_2_ on Fairbanks S(IV) formation focusing on major PM_2.5_ mass concentration events during extreme cold periods in 2020 (January 18-21) and 2022 (January 31 – February 3) is included in the supplemental material. The results all point to decreasing HCHO emissions to reduce HMS concentrations, whereas SO_2_ reductions could have a smaller impact on HMS. It follows then that the current focus on reducing SO_2_ and primary SO_4_^2–^ by lowering sulfur in residential heating oil^32^ may not substantially reduce HMS but would decrease S(VI) (SO_4_^2–^) concentrations, suggesting that HMS and other aldehyde-S(IV) adducts could become a larger fraction of PM_2.5_ sulfur species in Fairbanks if the focus is mainly on SO_2_ reductions. An increasing PM_2.5_ particle pH resulting from sulfur reductions could also produce a higher proportion of aldehyde-S(IV) adducts relative to sulfate. Wang et al.^27^ suggest a similar relative increase in HMS relative to SO_4_^2–^ over the NCP due air quality regulations aimed at reducing PM_2.5_ concentrations.

Overall, there are many factors that affect the concentrations of various S(IV) species in Fairbanks. High ALWC is more significant during periods of moderate temperatures and high RH, similar to what has been observed over the NCP,^27^ (where ALWC is exceptionally high relative to Fairbanks). However, our analysis shows that in Fairbanks the most important factor identified is the extreme low temperatures. Low temperature plays a key role in the heterogeneous chemistry of supercooled droplets by increasing the precursor gases’ Henry’s Law constants by orders of magnitude over those of more moderate temperatures (i.e., lowers gas volatility).^29^ Extreme low temperature also has a complex effect on particle pH, which modulates the concentrations of inorganic S(IV) species (R2 and R3), and therefore is also a critical parameter. Because of the complex behavior of PM_2.5_ pH in the extreme cold of Fairbanks winter and its potentially wide-ranging impact in cold environments in general, we present a detailed investigation of particle pH elsewhere.

## 4 Summary

Campbell et al.^29^ identified the presence of a large S(IV) peak in ion chromatographic (IC) measurements of wintertime PM_2.5_ in Fairbanks, Alaska. Based on a successful match with a Na-HMS standard, the peak was quantified and referred to as HMS, following the approach of other investigators, although it was recognized that other S(IV) species may co-elute with HMS. In that study, S(IV) species were found to be a substantial mass fraction relative to SO_4_^2–^ making it an important component of PM_2.5_ in Fairbanks winters. Here, we report on a more extensive Fairbanks air quality study in which we speciated the S(IV) by preconditioning aerosol filter samples extracted into water with H_2_O_2_ to separate the S(IV) IC peak into species unreactive with H_2_O_2_, referred to as HMS, and the remaining S(IV) species obtained by the difference, referred to as other S(IV). We found that by mass, PM_2.5_ HMS in Fairbanks ranges from 48 to 70 percent of the (total) S(IV), depending on the particle size range, implying that a significant fraction of what has been referred to as HMS in our past study^29^ is some other S(IV) component. A similar misidentification of S(IV) as exclusively HMS may apply to other studies.

Our analysis provides insight on the source and chemical form of the other S(IV). One possibility consistent with our observations is that a fraction of the other S(IV) is additional aldehyde-S(IV) adducts. These hydroxyalkylsulfonate (HAS) species are more complex aldehydes that form adducts with S(IV), much like HCHO forms HMS. They have been investigated and identified in cloud/fog water^12^ and observed (but not quantified) along with HMS in NCP haze.^24,50^ HAS species may exist as whole adducts in the particle but become unstable in our filter extraction and analysis process, resulting in identification of the adduct as dissociated inorganic S(IV) (i.e., other S(IV)). These species are known to be significantly less stable than HMS.^12^ We find that both HMS and the other S(IV) have similar size distributions, which are shifted to larger particle sizes relative to primary SO_4_^2–^ particles, suggesting a shared aqueous formation process. Moreover, the concentrations of both sets of species have a similar strong inverse temperature dependence. The effect of extreme low temperature is likely the cause for similar behaviors since it is a main driver of heterogeneous particle formation by raising precursor gas Henry’s Law constants and could also affect particle pH through reduced ammonia volatilization. The particle size distributions of cleaner periods differ from those of polluted cold periods, where coarse mode other S(IV) was at higher concentrations than HMS. Coarse mode other S(IV) could reflect the regional aged aerosol, or originate from local sources that impact the surface measurement site during cleaner periods of greater vertical mixing and dispersion.^31^ Past studies show S(IV) species from coal-fired power plants mainly in the form of physically adsorbed SO_2_, metal-S(IV) complexes and organic adducts in fly ash,^45^ although it is not clear that the more modern coal-fired power plants in Fairbanks would have similar emissions.

If S(IV) is mainly aldehyde-S(IV) adducts (HAS) (i.e., both HMS and other aldehyde-S(IV) adducts), it points to a combination of sources that produce these sulfur compounds in PM_2.5_; sulfur from mainly residential heating with fuel oil and lesser contributions from wood burning, and additional various sources of aldehydes, such as HCHO from vehicle emissions. Our analysis suggests that the aldehydes are rate limiting, implying that a focus on reductions in SO_2_ may increase HMS concentrations relative to S(VI).

Additional characterization techniques are required to identify the molecular form of the other S(IV) in the ambient particles. A further limitation with our method is possible sampling artifacts. Filter samples analyzed immediately after filter collection versus weeks following the experiment had higher concentrations. It cannot be ruled out that there may be loss of HMS, and especially other S(IV), in the time up to filter extraction and analysis. Artifacts could be minimized by applying the GT H_2_O_2_ flow injection system described here for offline S(IV) speciation to an online measurement method, such as a PILS. Higher time resolution could also provide additional insights over time-integrated filter samples. Further S(IV) speciation measurements in other locations with demonstrated high HMS levels and differing meteorological conditions, such as the NCP, would also provide additional ways to test hypotheses on the sources of various S(IV) species, their atmospheric chemical processing, and contributions to PM_2.5_ mass concentrations.

## Data Availability

Final data from the ALPACA study is available to the scientific community through the ALPACA data portal hosted by Arcticdata.io (https://arcticdata.io/catalog/portals/ALPACA) and for this paper at https://arcticdata.io/catalog/view/doi%10.18739%A2WP9T83H.

## Supporting Information Available

Discussion of relative role of HCHO and SO_2_ in HMS formation during severe cold pollution events; MOUDI cut sizes and particle diameter size range; summary of MOUDI sample collection periods and flow conditions; retention times for anion IC analysis using GT and UNH systems; quantification of HMS standard stability and resistance to oxidation by H_2_O_2_; quantification of bisulfite standard stability and removal by H_2_O_2_; decay of bisulfite peak area over time; decay of sulfite peak area over time; quantification of sulfite removal by H_2_O_2_; averages and statistical error of precursors, S(IV), HMS, sulfate, PM_2.5_, and temperature during 2020, 2021, and 2022 study periods; possible artifacts in PTR-ToF-MS measurements of aldehydes; diagram of filter storage and analysis process; investigation of bias at low concentrations of S(IV); S(IV) time series during 2020, 2021, and 2022 study period highlights; plots of HMS and normalized HMS vs. S(IV) and normalized S(IV); size distributions of sulfate and S(IV) in MOUDI samples; size distributions of HMS and other S(IV) in MOUDI samples; select size distributions with statistical analysis for mild, moderate, and severe pollution periods; ALWC vs. RH during the 2022 campaign; select aldehyde concentrations vs. formaldehyde; gas phase HCHO vs. SO_2_ during the 2022, colored by date; time series and S(IV)/sulfate vs. RH of major pollution events from 2020 and 2022 campaigns; S(IV)/sulfate vs. HCHO/SO_2_ during major pollution events from 2020 and 2022 campaigns; time series and ratio of gas phase HCHO and SO_2_ concentrations. (PDF)
