# Supplementary material for: Hydroxymethanesulfonate and Sulfur(IV) in Fairbanks Winter During the ALPACA Study
Source: ACS EST Air. 2024 May 15;1(7):646–59. doi: 10.1021/acsestair.4c00012 (PMC11250035; doi:10.1021/acsestair.4c00012)
Supplement: Supplementary file 1 — ea4c00012_si_001.pdf [file ea4c00012_si_001.pdf]

Supplemental Material

Hydroxymethanesulfonate and Sulfur (IV) in Fairbanks Winter During the ALPACA Study

Kayane Dingilian[1,a], Elliana Hebert[1], Michael Battaglia[1,b], James R. Campbell[2], Meeta Cesler-Maloney[2], William Simpson[2], Jason M. St. Clair[3], Jack Dibb[4], Brice Temime-Roussel[5], Barbara D'Anna[5], Allison Moon[6], Becky Alexander[6], Yuhan Yang[1], Athanasios Nenes[1,7,8], Jingqiu Mao[2*], Rodney J. Weber[1*]

[1] School of Earth and Atmospheric Sciences, Georgia Institute of Technology, Atlanta, Georgia 30332, USA

[2] Geophysical Institute and Department of Chemistry & Biochemistry, University of Alaska Fairbanks, Fairbanks, Alaska, 99775 USA

[3] Atmospheric Chemistry and Dynamics Laboratory, NASA Goddard Space Flight Center, Greenbelt, Maryland 20771, United States; Joint Center for Earth Systems Technology, University of Maryland Baltimore County, Baltimore, Maryland 21228, United States.

[4]Institute for the Study of Earth, Oceans, and Space, University of New Hampshire, Durham, New Hampshire, USA

[5]Aix Marseille Univ, CNRS, LCE, Marseille, France

[6]Department of Atmospheric Sciences, University of Washington, Seattle, Washington 98195, United States

[7]Laboratory of Atmospheric Processes and their Impacts, School of Architecture, Civil and Environmental Engineering, École Polytechnique Fédérale de Lausanne, Lausanne 1015, Switzerland

[8]Center for the Study of Air Quality and Climate Change, Institute of Chemical Engineering Sciences, Foundation for Research and Technology Hellas, Patras 26504, Greece

[a]Now at: Division of Chemistry and Chemical Engineering, California Institute of Technology

[b]Now at: U.S. Army DEVCOM CBC, Aberdeen Proving Ground, Maryland, 21010 USA

* Corresponding authors

S1. Relative Role of HCHO and SO_2 in HMS Formation During Extreme Cold Pollution Events

To investigate the relative roles of HCHO and SO_2 on Fairbanks HMS formation, and S(IV) species in general, we focus on major pollution events of high $PM_{2.5}$ mass concentration during an extreme cold event in 2020 (January 18-21) and 2022 (January 31 – February 3) (see F**igure S10a** for time series). Both events spanned approximately four days of temperatures in the -35 to -30°C range, and both had S(IV) peak concentrations in the range of 10 to 15 μg/m^3. Increased aerosol liquid water content ALWC does not appear to be driving the high S(IV) levels in these events because RH is negatively correlated with the mass ratio of S(IV)/sulfate **(Figure S10b**).

Ratios are used to reduce effects of temperature (and boundary layer effects) on emissions and dispersion of these species. **Figure S11** shows a trend of increasing S(IV)/sulfate ratio with increasing HCHO/SO_2 for the 2020 and 2022 cold events suggesting S(IV) formation relative to sulfate is limited by HCHO. We have shown that HMS is a significant fraction of S(IV) and is highly correlated during pollution (extreme cold) events of high $PM_{2.5}$ mass concentration, so S(IV) is used here as a surrogate for HMS to maximize the number of data points by using the PILS S(IV) instead of filter HMS data. The correlation shown in **Figure S11** is positive, but weak, which may reflect multiple factors that enhance the production of HMS (and therefore, S(IV)) relative to sulfate. **Figure S11** also shows data from the warm event at the end of Feb. 2022 (period (C) in **Figures 2, 3 and 7**). These data do not follow the trend of the two cold events (periods (A) and (B)) and there is no correlation between S(IV)/sulfate and HCHO/SO_2. The warm period was associated with higher HCHO concentrations (3.59 ppb vs. 2.84 ppb study average) and the HCHO/SO_2 ratio was higher (median 0.55) relative to the cold event of 2022 (median 0.19) possibly making formaldehyde an excess reactant. The time series of HMS, SO_2 and the ratio with the warm and cold events identified is shown in **Figure S12**. Vehicle emissions were higher in period (C) relative to other sources.[1] and correlations between HCHO and $PM_{2.5}$ black or elemental carbon also indicate HCHO mainly from vehicles. However, the observed positive correlations between S(IV)/sulfate vs HCHO/SO_2 are only observed during the cold events; for the overall study the correlation was r = - 0.18 implying it is not clear that HCHO as a rate limiting reactant applies for the entire campaign, although HCHO is almost always at lower concentrations then SO2 (**Figure S9** and **S12**).

Table S1. MOUDI impactor cut sizes (aerodynamic particle diameter, Dp, at 50% collection efficiency) at standard ambient temperature and pressure. In some cases, bottom stages were removed to achieve the desired 30 L/min sample flow rate measured at the beginning of the sampling.

Stage	Upper Dp Cut Size (µm)	Collected Dp Range (µm)
Inlet	18	> 18
1	10.0	10 - 18
2	5.6	5.6 - 10
3	3.2	3.2 - 5.6
4	1.8	1.8 - 3.2
5	1.00	1.00 - 1.8
6	0.56	0.56 - 1.00
7	0.32	0.32 -0.56
8	0.18	0.18 – 0.56
9	0.100	0.100 -0.18
After-Filter	0.100	0.10 – 0.010*

* For cases where a bottom stage was removed the After-Filter will measure all particles up to that lower size. For plotting and fitting the distributions the After-Filter is assumed to span sizes from the lower cut size of the impaction stage above down to a Dp of 0.01 µm.

Table S2. Summary of MOUDI samples collected during the ALPACA study. Flow rates were measured at the start and stop of the MOUDI measurement. At times, extreme cold resulted in lower flow rates at the end of the sampling periods. Concentrations were determined from the mean of the start and end flow rate. Cut sizes were not adjusted for deviations in flow rate from the desired 30 L/min shown in Table S1). The Sample ID is the month day (e.g., 0121 is Jan 21) at the end of the sample period). Times are local Alaska Standard Time (AKST). Size distributions are plotted in Fig. 5 for the three shaded samples.

Sample ID	Start Time	Start Flow, L/min	End Time	End Flow L/min	Flow Rate* Variability, %
M0121	1/17/2022 11:00	30	1/21/2022 8:56	26.3	13.1
M0124	1/21/2022 8:58	32	1/24/2022 9:17	30.39	5.2
M0126	1/24/2022 10:01	30.3	1/26/2022 9:16	32.558	7.2
M0130	1/26/2022 10:10	32.003	1/30/2022 9:04	26.89	17.4
M0201	1/30/2022 10:03	26.72	2/1/2022 9:04	16.626	46.6
M0203	2/1/2022 11:44	31.019	2/3/2022 10:06	11.343	92.9
M0207	2/3/2022 10:33	32.201	2/7/2022 9:11	30.034	7.0
M0211	2/7/2022 10:04	31.072	2/11/2022 9:14	30.106	3.2
M0215	2/11/2022 10:26	30.596	2/15/2022 9:04	29.763	2.8
M0219	2/15/2022 10:04	30.327	2/19/2022 9:03	30.839	1.7

* Flow rate variability is: Abs(start – stop flow)/avg(start, stop flow) 100%

Table S3. Various retention times for the Georgia Tech and University of New Hampshire IC analysis system used for the PILS, various bulk filters and MOUDI analysis.

Anion	GT Retention Times, min	UNH Retention Times, min
Chloride (Cl^-)	5.8	1.55
Nitrite (NO_2^-)	6.9	1.72
Bromide (Br^-)	8.7	2.50
Nitrate (NO_3^-)	10	2.60
S(IV) (HMS, HSO_3^-, SO_3^{2-})	15.6	4.00
Sulfate (SO_4^{2-})	16	4.55
Oxalate ($C_2O_4^{-2}$)	-	5.45

Table S4. Results on the stability of an HMS standard (NaHMS in solution) as a function of concentration due to conversion to sulfate during extraction and IC analysis for two different periods (columns 2 and 3), and during the H_2O_2 S(IV) speciation procedure (columns 4 and 5).

Sample HMS concentration mg/L (ppm)	% HMS loss Extraction/Analysis (Day 1)	% HMS loss Extraction/Analysis (Day 2)	% HMS loss Speciation (Day 1)	% HMS loss Speciation (Day 2)
0.4125	1.66	3.33	15.7	15.6
0.825	1.80	2.84	14.2	13.3
1.65	1.74	2.62	12.2	11.3
3.3	1.82	2.35	10.5	10.6

Percent loss is: (initial concentration – final concentration)/initial concentration x 100.

Table S5: Loss of bisulfite (HSO_3^- from 1% $NaHSO_3$ standard) due to conversion to sulfate during extraction and IC analysis with the GT system (column 2), and from GT method of H_2O_2 treatment for different starting concentrations of HSO_3^- (column 3).

Sample HSO_3^- Concentration, mg/L (ppm)	% HSO_3^- loss Extraction/Analysis	% HSO_3^- loss removed by H_2O_2
0.25	14.1	94.68
0.50	13.4	97.66
1.00	12.6	98.99
2.00	11.0	99.25

Percent loss is: (initial concentration – final concentration)/initial concentration x 100.

Table S6. Tests on the stability of bisulfate (HSO_3^-) in the extract solution. Data show loss of HSO_3^- (HSO_3^-, starting with 2 mg/L (ppm) in DI water) in solution as measured by the decay of the S(IV) IC peak over different time intervals.

Elapsed Time, min	S(IV) Peak Area Loss (%)
Start (0)	N/A
37	1.68
67	3.23
75	4.40
142	4.56

Table S7: Tests on the stability of sulfite (SO_3^{2-}) in the extract solution. Data show loss of SO_3^{2-} (SO_3^{2-} starting concentration of 750 mg/L (ppb) in DI water) solution as measured by the decay of the S(IV) IC peak.

Time	S(IV) Peak Area Loss (%)
Start (0)	N/A
22 min	3.97
41 min	6.85
2 h 9 min	22.1
4 h 38 min	32.6

Table S8: Removal efficiency of sulfite as measured by S(IV) peak area by H_2O_2 after 10 minutes.

Concentration mg/L (ppm)	initial area	area at 10 min	% removed
0.21	0.0676	0.0074	89.1
0.42	0.1659	0.0189	88.6
0.83	0.3882	0.0428	89.0
1.67	0.8411	0.0927	89.0
3.33	1.961	0.1919	90.2

Table S9. Summary (means ± stdev) of various $PM_{2.5}$ species, gas phase precursors and meteorological parameters for three consecutive measurements in wintertime Fairbanks. Data for periods of pollution events of high $PM_{2.5}$ mass concentration are also shown, denoted by a subscript E (for events).

	2020	2021	2022
Study Period	Jan 18 – Mar 7	Jan 7 – Feb 21	Jan 17 – Feb 26
Event Period	Jan 18* – Jan 20	Jan 11 – Jan 14	Jan 31 – Feb 3
S(IV), $\mu g/m^3$	0.32 ± 0.98	0.32 ± 0.65	0.38 ± 0.88
HMS**, $\mu g/m^3$	-	-	0.22 ± 0.41***
Sulfate, $\mu g/m^3$	1.68 ± 1.23	1.57 ± 1.55	2.70 ± 2.46
S(IV)/Sulfate (mol/mol)	0.11 ± 0.21	0.11 ± 0.14	0.07 ± 0.13
$S(IV)_E$, $\mu g/m^3$	2.62 ± 2.91	1.01 ± 1.44	1.99 ± 1.73
HMS_E**, $\mu g/m^3$	-	-	1.20 ± 0.52***
$Sulfate_E$, $\mu g/m^3$	2.72 ± 1.67	3.51 ± 2.26	7.81 ± 2.53
S(IV)/$Sulfate_E$ (mol/mol)	0.70 ± 0.43	0.19 ± 0.18	0.21 ± 0.15
$PM_{2.5}$, $\mu g/m^3$	13.16 ± 10.02	22.47 ± 10.62	11.54 ± 8.56
$PM_{2.5,E}$, $\mu g/m^3$	26.15 ± 12.61	32.83 ± 12.55	31.18 ± 8.17
SO_2, ppbv	6.63 ± 5.76	10.57 ± 6.86	25.66 ± 17.32
$SO_{2,E}$, ppbv	17.13 ± 3.90	13.74 ± 4.84	52.29 ± 13.06
HCHO, ppbv	2.66 ± 7.18	-	2.84 ± 1.73****
$HCHO_E$, ppbv	3.38 ± 1.39	-	4.46 ± 1.93****
T, °C	-21.52 ± 8.25	-17.89 ± 7.17	-17.40 ± 7.33
T_E, °C	-30.81 ± 1.34	-15.25 ± 4.93	-29.00 ± 1.09

* Data collection in 2020 appears to commence in the middle of or shortly before the start of its main pollution event; it is uncertain which day/time the event began

** in Campbell et al.[2] what is referred to as HMS actually was S(IV) so we use the term S(IV) in this study when citing the results from Campbell et al.

*** Calculated via GT PM2.5 filters with H_2O_2 treatment, data from 1/24/22 onwards

**** HCHO data incomplete for 2022, starts on 1/30

Table S10. $PM_{2.5}$ mass concentration determined by averaging ADEC hourly measurements to the MOUDI sampling time and concentrations of sulfur species for the fine (particle aerodynamic diameter, Dp, < 3.2 µm) and coarse modes (Dp 3.2 µm and larger) by integrating over the MOUDI size ranges. All concentrations are in µg/m^3.

Sample ID	ADEC $PM_{2.5}$ Mass AVG	Fine HMS	Fine Other S(IV)	Fine S(IV)	Fine SO_4^{2-}	Coarse HMS [3]	Coarse Other S(IV)	Coarse S(IV)	Coarse SO_4^{2-}
M0121	14.4043	0.1704	0.1991	0.3694	4.8280	0.0285	0.0918	0.1203	0.2340
M0124	10.6849	0.0955	0.0648	0.1603	1.8205	0.0141	0.0406	0.0547	0.1387
M0126	6.4468	0.0348	0.0252	0.0600	1.3348	0.0201	0.0668	0.0869	0.1790
M0130	15.3789	0.1281	0.1070	0.2350	3.5989	0.0185	0.0267	0.0452	0.2135
M0201	33.7660	0.5410	0.3844	0.9254	4.1922	0.0159	0.0069	0.0229	0.1363
M0203	29.6170	1.1867	0.6568	1.8435	7.6664	0.0431	0.0809	0.1240	0.1349
M0207	7.5789	0.0500	0.0993	0.1492	1.9884	0.0246	0.0627	0.0874	0.0827
M0211	5.9684	0.0507		0.0507	1.3169	0.0132		0.0132	0.0748
M0215	7.3404	0.1280		0.1280	1.6391	0.0516		0.0516	0.0711
M0219	6.2000	0.0231	0.0233	0.0465	1.7586	0.0130	0.0212	0.0341	0.0692

Table S11. Possible interferences in PTR-ToF-MS measurements of various aldehydes.

VOC	Ion Formula (RH^+)	Ion exact mass	Possible interferences	Experimental LOD as 3xσ (ppb)
Acetaldehyde	$C_2H_4OH^+$	45.033	Ethylene glycol[a]	0.012
Glycoladehyde	$C_2H_4O_2H^+$	61.028	Acetic acid, Ethylacetate fragment, peroxy acetic acid fragment, methyl acetate[b],	0.030
Methyl glyoxal	$C_3H_4O_2H^+$	73.028	Acrylic acid[c]	0.003
Benzaldehyde	$C_7H_6OH^+$	107.037		0.002

a [3], b [4], c [5]

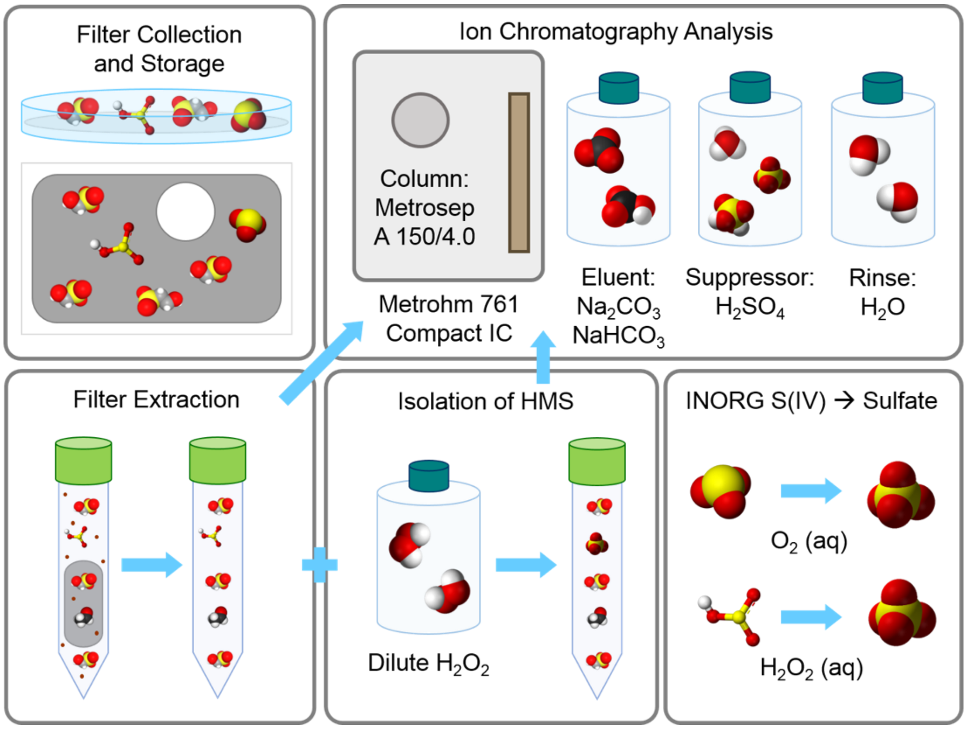

Figure S1. Diagram of filter storage and analysis process. Top left: Filters were stored in sealed plastic petri dishes at -20°C until analysis. Either whole filters, (GT $PM_{2.5}$ or MOUDI), or punches taken at random locations on the filter (UW high volume sampler), were used for extraction and analysis. Bottom left: Extraction in milli-Q water was done in 15 mL centrifuge tubes. Samples from filter extractions were split into two aliquots. Top right: One portion of the extract was directly analyzed by the anion IC (GT system is illustrated) to obtain S(IV) and other anion concentrations in the extract. All S(IV) species elute from the column at the same time. To separate out HMS, (Bottom Middle), a second portion of the extract was combined with diluted H_2O_2 and that sample analyzed by the same anion IC system. Bottom Right: In the H_2O_2 treatment, HMS is largely unaffected whereas other S(IV) species, such as inorganic S(IV) is converted to sulfate. The IC measurement after the H_2O_2 treatment is then largely as just HMS.

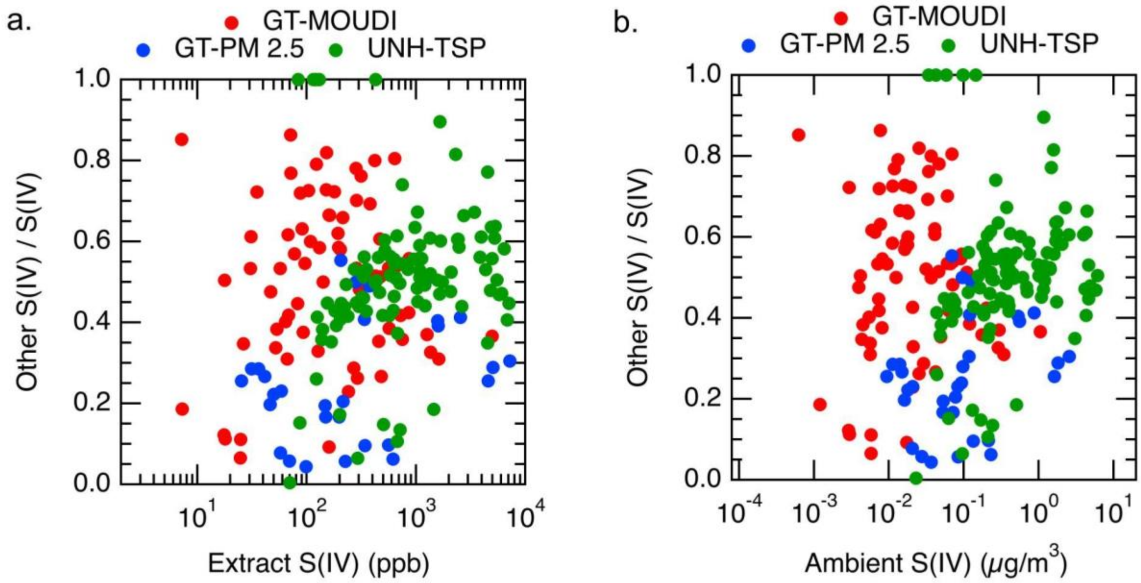

Figure S2. Assessment of the conversion of HMS to sulfate in the GT and UNH H_2O_2 treatment systems as a function of extract liquid concentration by comparing different filter sampling systems. Other S(IV) is S(IV) – HMS. Preferential conversion of HMS to sulfate at lower concentrations in S(IV) would lead to a bias of higher Other S(IV)/S(IV) with lower S(IV). No evidence of this is seen in the plots above.

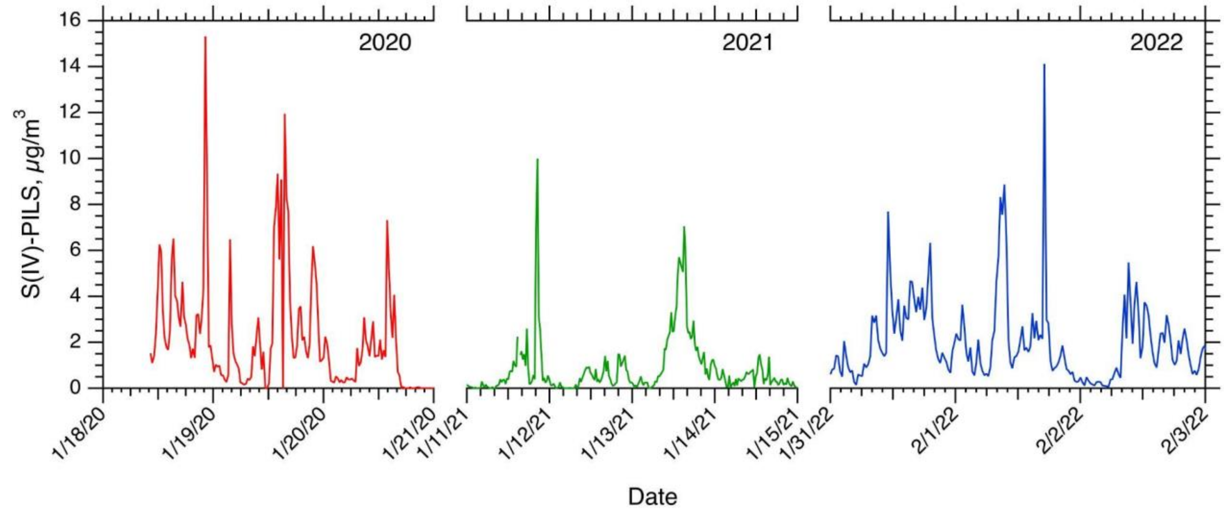

Figure S3: Measurements of S(IV) with the PILS-anion IC during events of high S(IV) in three consecutive winters in Fairbanks, Alaska.

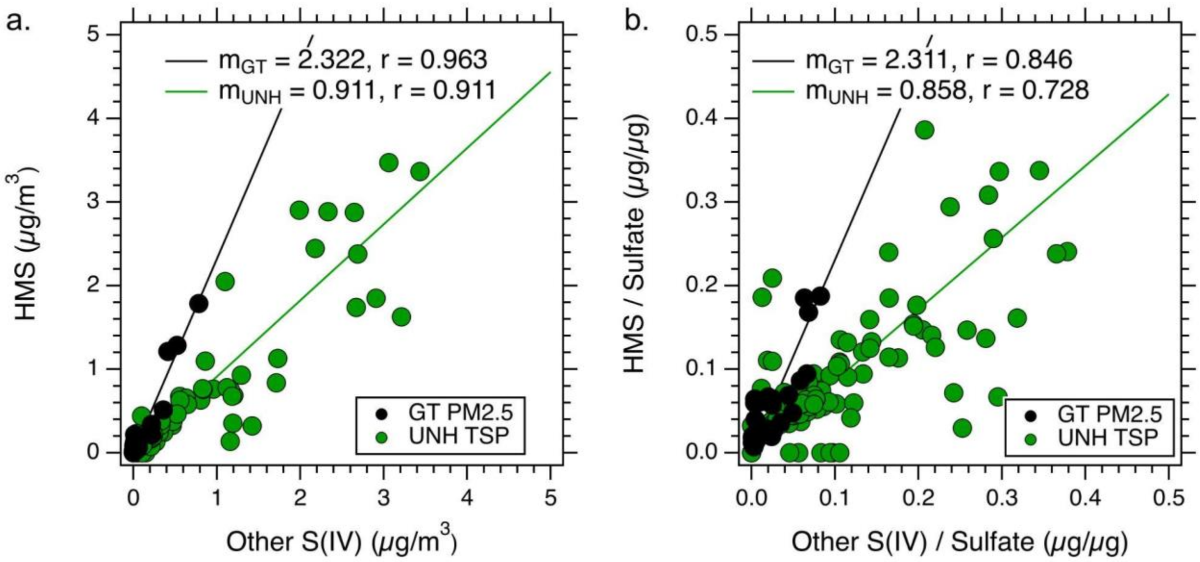

Figure S4: Comparison of (a) HMS and other S(IV) and (b) each relative to sulfate for $PM_{2.5}$ and TSP filter samples.

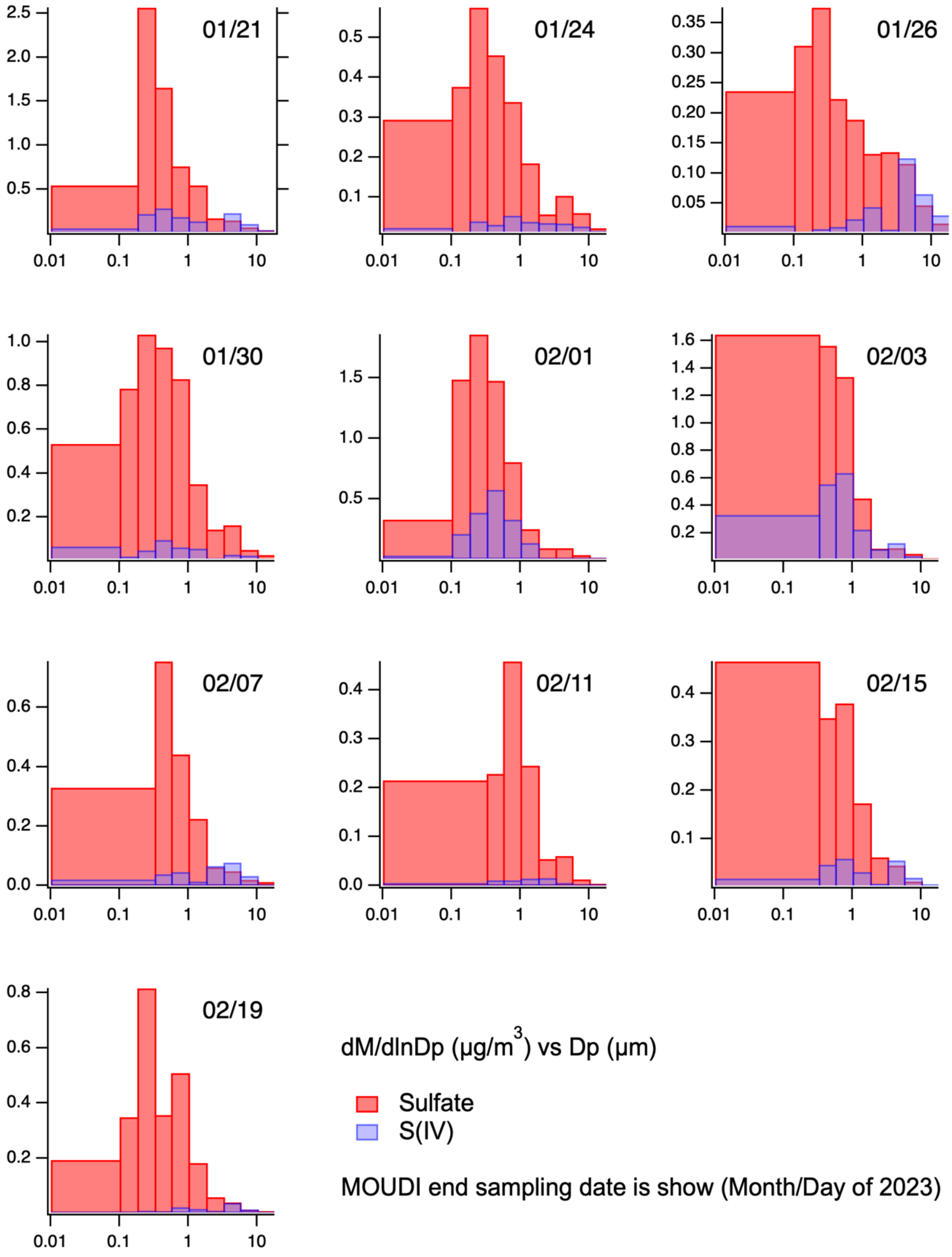
01/21
01/24
01/26
01/30
02/01
02/03
02/07
02/11
02/15
02/19
0.01
0.1
1
10
dM/dlnDp (µg/m³) vs Dp (µm)
Sulfate
S(IV)
MOUDI end sampling date is show (Month/Day of 2023)

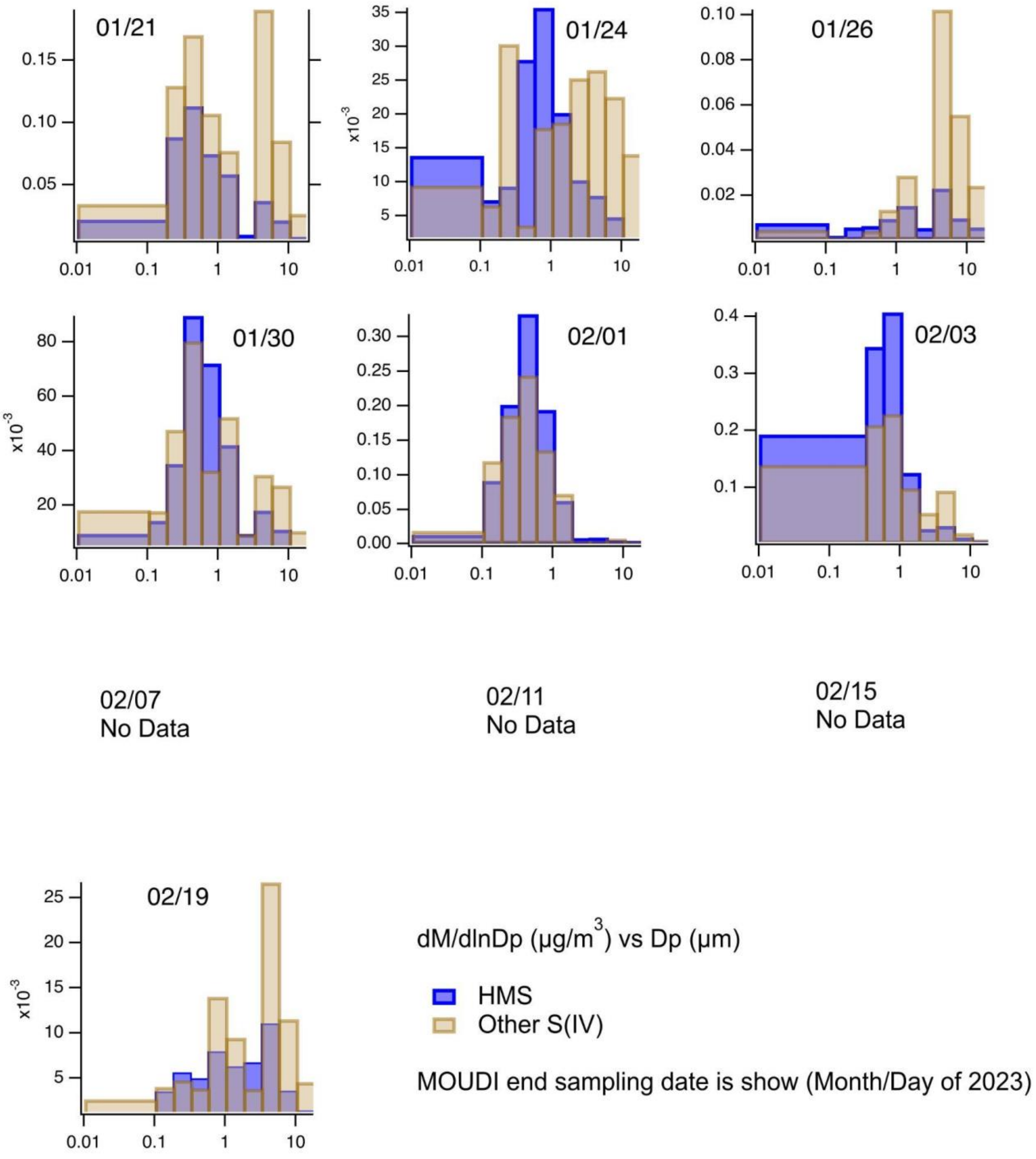

Figure S5: All MOUDI data for the ALPACA study. No data for the HMS and other S(IV) analysis resulted from too low of concentrations for the S(IV) speciation analysis. Other S(IV) is the difference in S(IV) and HMS.

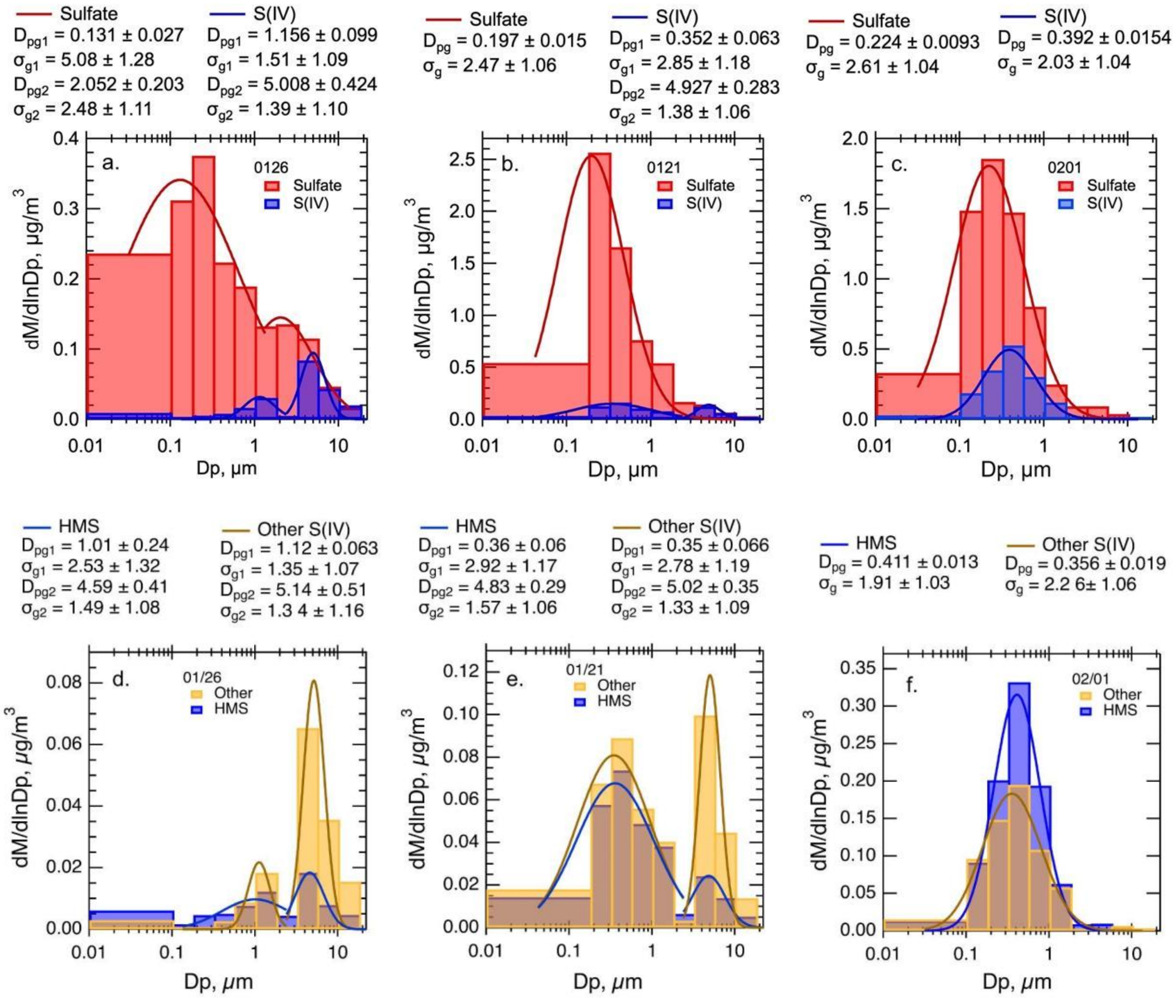

Figure S6: Selected size distributions for 2-day average MOUDI measurements during the ALPCA study shown in Figure 5. Data for lognormal fits for various modes are tabulated above each plot. The uncertainty with the fits is 1 standard deviation. Other S(IV) is S(IV)-HMS.

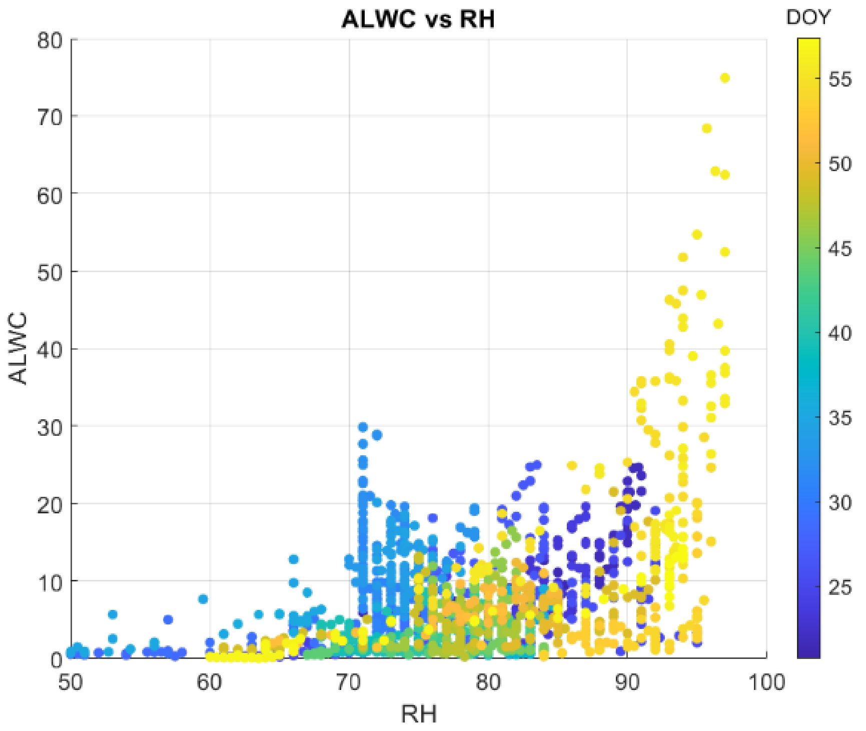

Figure S7: Aerosol liquid water content (ALWC in µg/m^3) vs. RH (in %) for the duration of the 2022 ALPACA campaign colored by date determined with the thermodynamic model ISORROPIA.

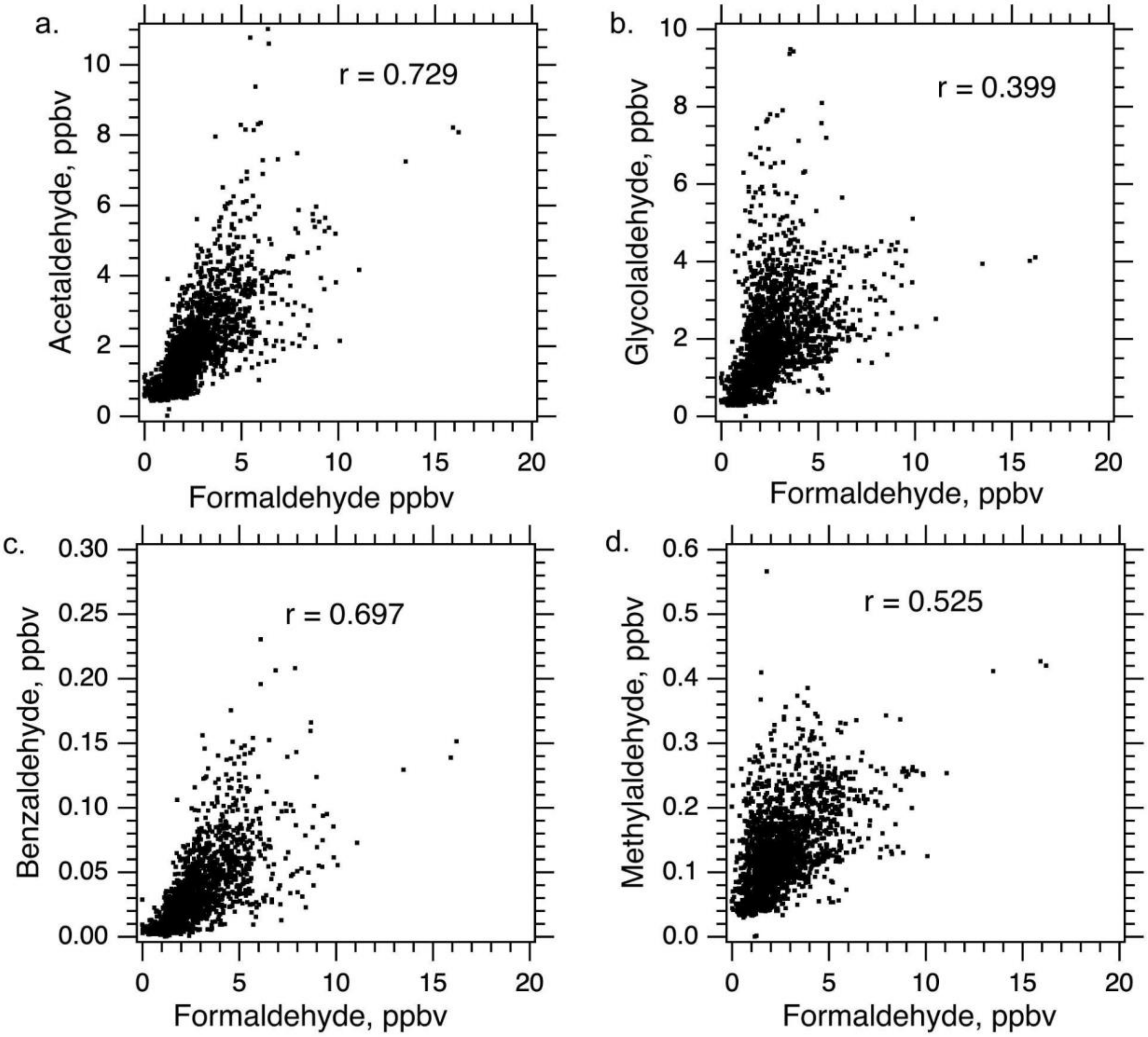

Figure S8. Comparisons between AERIS-COFFEE measured formaldehyde and other aldehydes measured via PTR-ToF-MS during the ALPACA study. The Pearson correlation coefficient is shown in each plot. Also see Table 1.

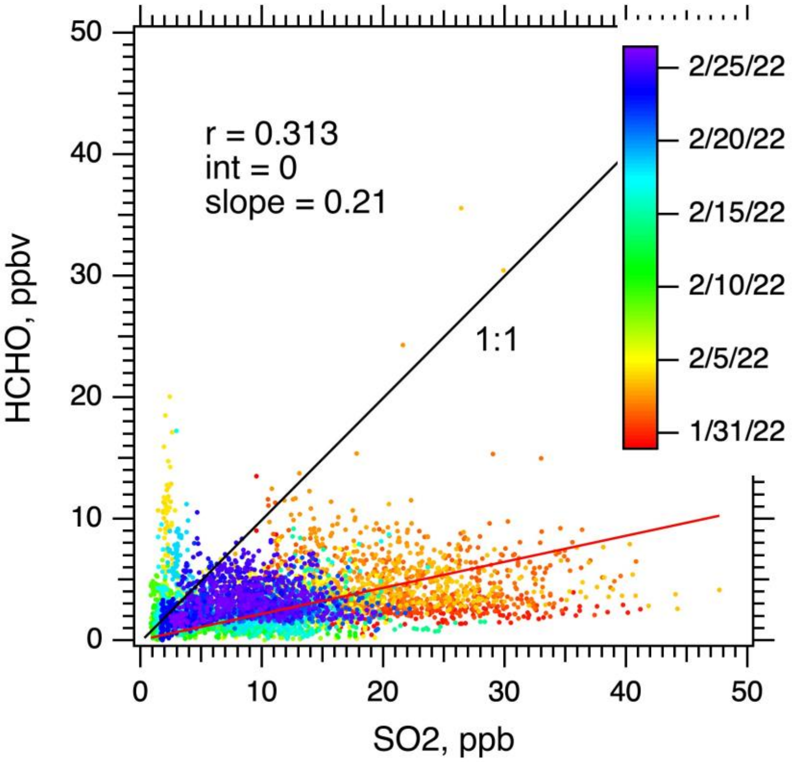

Figure S9. Comparison between gas phase HCHO and SO_2 concentrations for all data collected during the ALPACA field study in Fairbanks. Data points are colored by date based on AKST.

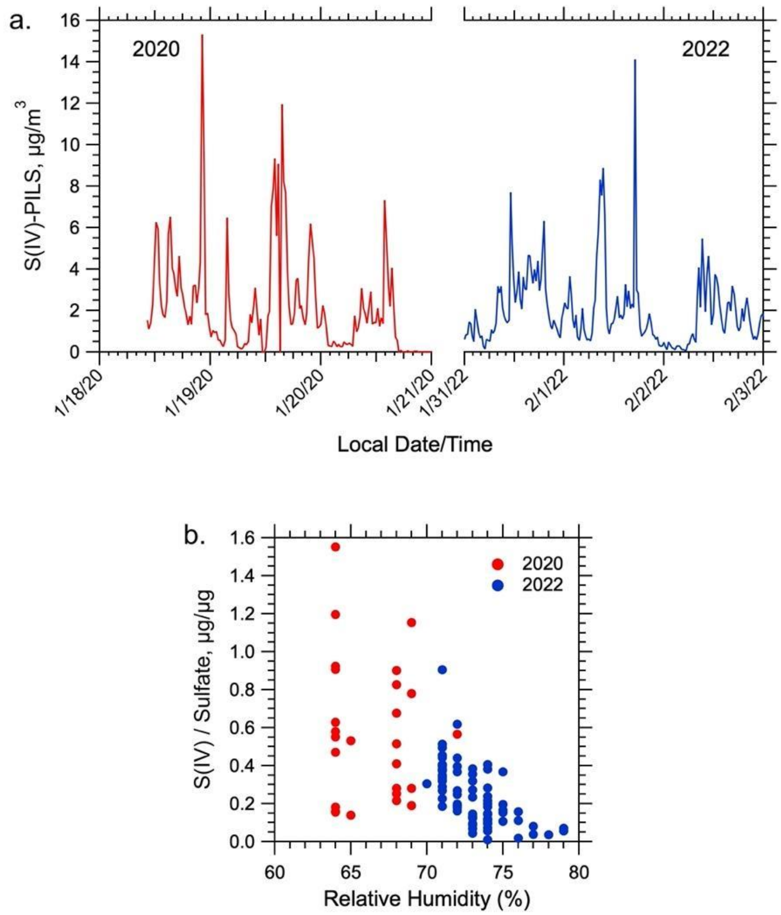

Figure S10: (a) Time series of major pollution events of high $PM_{2.5}$ mass concentration from the 2020 and 2022 campaigns during extreme cold periods. (b) Mass ratio of $PM_{2.5}$ S(IV) to sulfate versus relative humidity.

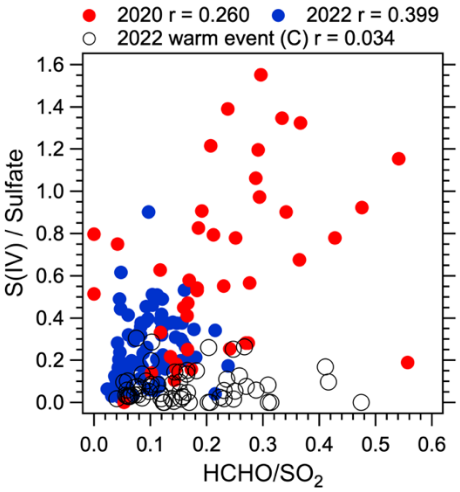

Figure S11. Relationship between mass ratios of $PM_{2.5}$ S(IV) to sulfate and gas phase HMS precursors formaldehyde (HCHO) to sulfur dioxide (SO_2) during a cold event of 2020 and 2022 (B in **Fig. 2**), and warm event at the end of 2023 (C in **Fig. 2**).

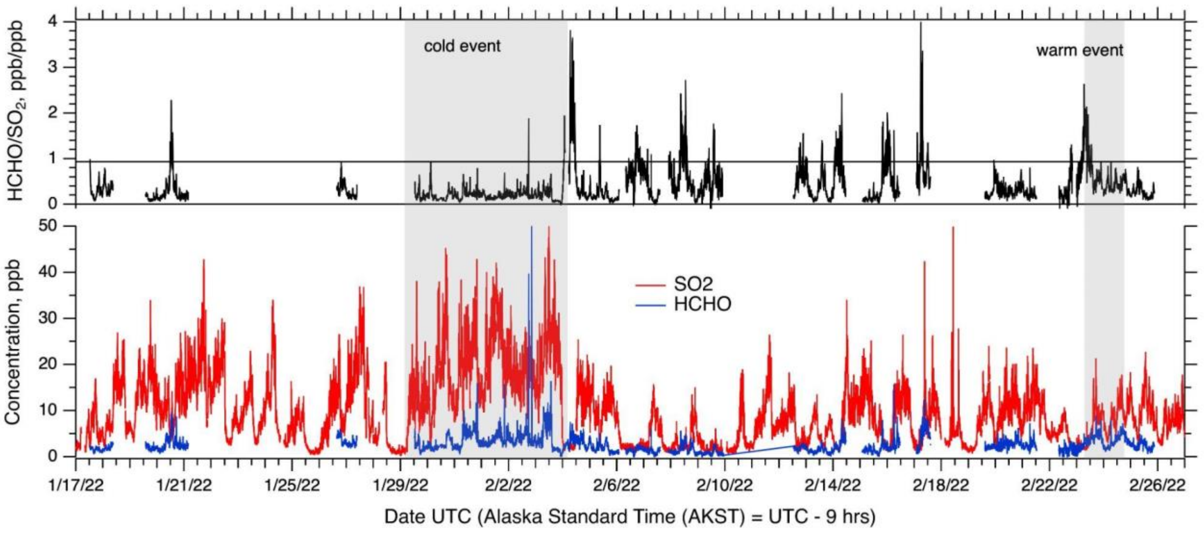

Figure S12. Time series of HCHO and SO_2 concentrations and the ratio. The cold and warm events are identified.

References

(1) Yang, Y.; Battaglia, M. A.; Mohan, M. K.; Robinson, E. S.; DeCarlo, R. F.; Edwards, K. C.; Fang, T.; Kapur, S.; Shiraiwa, M.; Cesler-Maloney, M.; et al. Assessing the Oxidative Potential of Outdoor PM2.5 in Wintertime Fairbanks, Alaska. *Environ. Sci. Tech. Air* **2024**, *1* (3), 175-187. DOI: https://doi.org/10.1021/acsestair.3c00066.
(2) Campbell, J.; Battaglia, M.; Dingiliian, K.; Cesler-Maloney, M.; Clair, J. S.; Hanisco, T.; Robinson, E.; DeCarlo, P.; Simpson, W.; Nenes, A.; et al. Source and Chemistry of Hydroxymethanesulfonate (HMS) in Fairbanks, Alaska. *Envron. Sci. Technol.* **2022**, *56* (12), 7657-7667. DOI: 10.1021/acs.est.2c00410.
(3) Wood, E. C.; Knighton, W. B.; Fortner, E. C.; Herndon, S. C.; Onasch, T. B.; Franlkin, J. P.; Worsnop, D. R.; Dallmann, T. R.; Gentner, D. R.; Goldstein, A. H.; et al. Ethylene Glycol Emissions from On-road Vehicles. *Environ. Sci. Tech.* **2015**, *49* (6), 3322-3329. DOI: doi.org/10.1021/acs.est.5b00557.
(4) Baasandorj, M.; Millet, D. B.; Hu, L.; Mitroo, D.; Williams, B. J. Measuring acetic and formic acid by proton-transfer-reaction mass spectrometry: sensitivity, humidity dependence, and quantifying interferences. *Atmos. Meas. Tech.* **2015**, *8* (3), 1303-1321. DOI: doi.org/10.5194/amt-8-1303-2015.
(5) Koss, A. R.; Sekimoto, K.; Gilman, J. B.; Selimovic, V.; Coggon, M. M.; Zarzana, K. J.; Yuan, B.; Lerner, B. M.; Brown, S. S.; Jimenez, J. L.; et al. Non-methane organic gas emissions from biomass burning: identification, quantification, and emission factors from PTR-ToF during the FIREX 2016 laboratory experiment. *Atmos. Chem. Phys.* **2018**, *18* (5), 3299-3319. DOI: 10.5194/acp-18-3299-2018.
